# Pathogenic convergence of CNVs in genes functionally associated to a severe neuromotor developmental delay syndrome

**DOI:** 10.1186/s40246-021-00309-4

**Published:** 2021-02-08

**Authors:** Juan L. García-Hernández, Luis A. Corchete, Íñigo Marcos-Alcalde, Paulino Gómez-Puertas, Carmen Fons, Pedro A. Lazo

**Affiliations:** 1 Molecular Mechanisms of Cancer Program, Instituto de Biología Molecular y Celular del Cáncer, Consejo Superior de Investigaciones Científicas (CSIC), Universidad de Salamanca, Salamanca, Spain; 2 Instituto de Investigación Biomédica de Salamanca (IBSAL), Departamento de Hematología, Hospital Universitario de Salamanca, Salamanca, Spain; 3 Network Center for Biomedical Research in Cancer (CIBERONC), Salamanca, Spain; 4 Centro de Biología Molecular Severo Ochoa, CSIC-Universidad Autónoma de Madrid, Cantoblanco, Madrid, Spain; 5 Biosciences Research Institute, School of Experimental Sciences, Universidad Francisco de Vitoria, Pozuelo de Alarcón, Madrid, Spain; 6 Neurology Department, Hospital Sant Joan de Déu, Sant Joan de Déu Research Institute, Esplugues de Llobregat, Barcelona and CIBERER, Instituto de Salud Carlos III, Barcelona, Spain

**Keywords:** CNV, Variome, Dystonia, Cerebral palsy, Neuromotor delay, Epilepsy

## Abstract

**Background:**

Complex developmental encephalopathy syndromes might be the consequence of unknown genetic alterations that are likely to contribute to the full neurological phenotype as a consequence of pathogenic gene combinations.

**Methods:**

To identify the additional genetic contribution to the neurological phenotype, we studied as a test case a boy, with a *KCNQ*2 exon-7 partial duplication, by single-nucleotide polymorphism (SNP) microarray to detect copy-number variations (CNVs).

**Results:**

The proband presented a cerebral palsy like syndrome with a severe motor and developmental encephalopathy. The SNP array analysis detected in the proband several de novo CNVs, nine partial gene losses (*LRRC55*, *PCDH9*, *NALCN*, *RYR3*, *ELAVL2*, *CDH13*, *ATP1A2*, *SLC17A5*, *ANO3*), and two partial gene duplications (*PCDH19*, *EFNA5*). The biological functions of these genes are associated with ion channels such as calcium, chloride, sodium, and potassium with several membrane proteins implicated in neural cell-cell interactions, synaptic transmission, and axon guidance. Pathogenically, these functions can be associated to cerebral palsy, seizures, dystonia, epileptic crisis, and motor neuron dysfunction, all present in the patient.

**Conclusions:**

Severe motor and developmental encephalopathy syndromes of unknown origin can be the result of a phenotypic convergence by combination of several genetic alterations in genes whose physiological function contributes to the neurological pathogenic mechanism.

**Supplementary Information:**

The online version contains supplementary material available at 10.1186/s40246-021-00309-4.

## Background

Children with severe non-hereditary neurodevelopmental delay, and neonatal or early onset of epileptic seizures, recently named as developmental and epileptic encephalopathy [1, 2], frequently have an unknown etiology, which are likely to be very heterogeneous. In this context, genetic alterations can play a relevant pathogenic role. Genomic studies based on whole-exome sequencing (WES) and single-nucleotide polymorphism (SNP) microarray to detect CNVs can identify genetic alterations contributing to the pathogenic mechanism of these complex clinical phenotypes [3]. These underlying genetic alterations may be of different types, and the better known are those associated to dominant point mutations. However, the genetic heterogeneity of complex neurological diseases such as cerebral palsy [4], epilepsy [5], and autism [6, 7] has already been associated to a combination of several different pathogenic gene variants, and to several CNVs in affected individuals, and the combination of CNV changes contributes to the individual variome [8, 9]. CNVs are structural gene alterations deleting or duplicating gene segments. Genomic SNP microarray studies have detected multiple CNV changes, deletions, and duplications, which are consistent with the heterogeneity observed in epileptic patients [10], but no common pattern was identified. The presence of a CNV in a gene can either alter its level of expression, the stability of the RNA, the structure of the protein, or its subcellular localization. Any of these effects will alter their function, which may be even more relevant in proteins associated to the membranes where they can play a role in specific neural functions, ion and neurotransmitter transport, neural cell interactions, or signal transmission.

Neonatal onset epilepsies related to *KCNQ2* mutations share a loss of the potassium channel function. However, a common loss of function can cause very heterogeneous neurological phenotypes that accompany the epileptic phenotype [11, 12]. Therefore, it is likely that there are additional unidentified genetic changes that can contribute to the heterogeneity and complexity of the individual neurological phenotype [5, 10, 12]. An alternative approach to detect candidate pathogenic genes is to search for specific CNV alterations in a proband with respect to the family members. Moreover, the analysis of CNVs in these patients has detected a variable number of additional genetic changes, but did not identify a specific or unique pattern of specific gene alterations common to different patients [13]. In neurons, where ion channels and specific cellular interactions are critical for its development and correct functions, CNVs can alter protein level, and their subcellular localization or density. These CNVs can play a pathogenic role if associated to the neurological functions that are altered in the clinical phenotype. The identification of underlying genetic alterations in neuromotor developmental disorders is proving very useful in a significant number of neurological phenotypes [14–16].

Human normal diversity is a consequence of different combinations of non-pathogenic gene variants. Therefore, it is likely that heterogeneity in neurological phenotypes can also be a consequence of alternative combinations of genetic changes in genes associated to cellular functions that are involved in the phenotype and constitute the individual variome [8, 9]. Therefore, we tested this hypothesis by searching for CNV variome differences among the four family members of a complex case of a child, as test case, that presented a developmental and epileptic encephalopathy of neonatal onset that has a novel *KCNQ2* mutation resulting from a partial KCNQ2 exon 7 duplication that impairs its inhibitory signal [17] and to determine if the proband has a CNV pattern associated to neuronal functions. However, the complexity of the neurological syndrome suggested that, although this mutation is necessary, there has to be additional cooperating genetic alterations. We have approached the identification of the underlying genetic problem by studying CNV alterations and their association to genes whose function can contribute to the neuropathological phenotype reported in the proband and absent in the family [17], as an alternative approach to searching for common variants among unrelated individuals with similar phenotypes. Complex neurodevelopmental delay and epileptic phenotypes might be the result of de novo combination of CNV alterations in genes associated to neurological functions that contribute to the patient syndrome and determine its pathogenic mechanism.

## Materials and methods

### Standard protocol approvals, registrations, and patient consents

The genomic studies were performed for diagnosis of a pediatric neurological syndrome of unknown etiology. For the diagnostic genomic studies, total DNA was obtained from peripheral blood samples. Written informed consent for the genomic study was obtained from both parents. Research protocol and consent forms were approved by the Institutional Review Boards of Hospital Universitario de Salamanca-Centro de Investigación del Cancer as reported in a previous publication [17].

### SNP microarray

The SNP microarray analysis of genomic alteration was performed using the matrix chip CytoScan HD array (Affymetrix; Thermo Fisher Scientific, Inc.) following manufacturer’s instructions. The matrix contains 2.696.550 probes that include 743,304 SNPs and 1,953,246 non-polymorphic probes. The mean spacing between probes for RefSeq genes is 800 bp, and 96% of the genes are represented. Briefly, 500 ng of DNA was digested with Nsp1 for 2 h at 37 °C. Digested DNA was purified and ligated to primers/adaptors at 16 °C for 15 h. The products of this ligation were used to generate amplicons by PCR using the primers provided by the manufacturer (Affymetrix). The PCR program was one cycle at 94 °C for 1 min, thirty-five cycles (94 °C for 30 s, 60 °C for 45 s, and 65 °C for 1 min) and a final extension cycle at 68ªC for 7 min. Purified PCR products were digested with DNase I for 35 min at 37ªC, and the fragmented DNA was labeled with biotinylated nucleotides with deoxynucleotide terminal transferase for 4 h at 37ªC. A 250 μg of fragmented DNA was hybridized to the Chip Affymetrix chip Cytoscan HD preequilibrated at 50 °C for 18 h. The matrixes were washed and stained in the GeneChip Fluidics Station 450 (Affymetrix Inc.), and DAT images were acquired with the GeneChip Scanner 3000 7G (Affymetrix Inc.). The data files (archives.cel) were generated with the Affymetrix GeneChip Command Console *Software* (*AGCC*) software (Affimetrix Inc., Santa Clara, CA). Data analysis was performed using the analysis program package from Affymetrix Chromosome Analysis Suite (CHAS 4.0). The CGH results were compared with data in ChAs 3.3 NetAffix Build 33.2(Hg19) as reference. In addition, the aCGH results were compared among the four members of the family to confirm their presence in the proband, but not in the parents and sister. Furthermore, the exome assay segments were determined through the Control-Freec program [18], using as reference either the father, mother, and daughter exomes. An example that that shows the overlapping alterations (nCN-LOH, gains, and losses) between the aCGH microarray and WES exome assays for the patient with respect to the other three family members is represented in Supplementary Figure S1. Graphs were depicted through the custom table option in the UCSC Genome Browser for the GRCh37 human genome version.

### Whole exome sequence and comparison with SNP microarray

The WES study has been previously published in the report with the *KCNQ2* exon 7 partial duplication [17]. CN variant analysis in WES data was performed using VarScan2 [19].

Briefly, raw FASTQ files were mapped against the hg19 version of the human genome using the BWA-MEM aligner. The resulting BAM files were pre-processed following the GATK [20]: workflow, marking PCR duplicates, and correcting errors in the base quality scores. The exome assay CNV segments were determined through both the Control-Freec [18] and the VarScan2 [19] programs, using as a reference either the father, mother, or daughter exomes. The Integrative Genomics Viewer (IGV) [21] was used to inspect the sequencing data and construct the graphs associated to WES-CNV analysis.

### Analysis of gene alterations and function associated to clinical phenotypes

All genes alterations or variants detected by either SNP microarray or WES were analyzed using the VarElect program [22] (LifeMap Sciences Inc., Tel Aviv, Israel), to search for a correlations between the biological function of altered genes and the clinical phenotypes of the case, in order to detect a mechanistic implication of the gene contribution to clinical symptoms.

## Results

### Neuromotor developmental delay phenotype of patient

The patient is a 6-year-old boy presenting a cerebral palsy-like syndrome associated to severe development delay of unknown origin. The patient has a severe axial hypotonia without head control, spastic-dystonic tetraparesis, and peripheral neuro-axonal motor neuropathy, hypertonia of all limbs with dystonic movements of arms, no hand use and is not able to sit or crawl, and is unable to talk. He also presents an epileptic encephalopathy of neonatal onset with seizures well controlled since 4 years of age [17]. The full clinical study has already been reported [17]. The WES study detected a partial KCNQ2 exon 7 duplication (Clinvar ID 617505) that impairs its function [17], but it did not identify any other neuropathogenic mutation or gene variant [17] that could be functionally associated to the complex neurological phenotype of this patient.

### Cooperating CNVs in neuro-pathogenic genes

We hypothesized that the proband might have additional candidate genetic alterations, which must occur in genes associated to neurological functions, and if they are known to have mutations, these mutants should also be associated to neurological phenotypes. Therefore, the phenotypic convergence is due to a combination of several alterations in genes whose protein biological functions can contribute to different aspects of this complex neurological syndrome. In this context, we reasoned that additional genetic factors have to contribute to this complex clinical phenotype, either in the form of additional genetic mutations or changes in gene copy number that will alter the expression and level of their proteins. Because the WES study did not identify any additional gene variant or mutant associated to the pathology, in addition to the known KCNQ2 exon 7 partial duplication [17], and in order to detect additional cooperating gene alterations that contribute to the pathogenesis of the patient complex neurological syndrome, the genome of the proband and family members was further studied by SNP microarrays to detect CNVs.

The SNP microarray study of the CNV variome in the patient can detect changes genes related to the altered neurological functions, and therefore, genes located in them are candidates to be involved in the clinical phenotype. The number of CNVs was similar in the four members of the family (Table 1), most of them were already known, and a small proportion of them were de novo CNVs; however, they were not shared among the four family members. The microarray study detected in the proband, compared to the other three family members, several genomic de novo CNVs larger than 3 kb (Table 2), and its markers are detailed in the Supplementary Table S1. Larger de novo loss of heterozygosity (LOH) genomic regions in the patient are detailed in Supplementary Table S2. The genes included within these de novo CNVs unique to the proband child are indicated in Supplementary Tables S1 and S2. In five genes, there is a deletion affecting several exons (*SLC17A5*, *RYR3*, *ATP1A2*, *ELAVL2*, *ANO3*), one gene (*PCDH19*) has an exon duplication, three genes have intronic deletions (*NALCN*, *CDH13*, *LRCC55*), and two genes have an intronic duplication (*EFNA5*, *PCDH9*). Intronic alterations can alter the processing of the RNA or its stability.

**Table 1:** Genetic changes detected by aCGH in the family. The complete list of CNVs detected in the patient are shown in supplementary Table S2 including its markers (rs identifiers), size, and chromosomal position. The CGH results were compared with data in ChAs 3.3 NetAffix Build 33.2(Hg19) as reference. Known means that they have already been detected in other unrelated individuals

*De novo* CNV alterations in each family member							
	Small CNV (1-3kbp)						Large CNV losses (1–2 Mbp)
	CNV losses (autosomes/sex chrom)			CNV gains (autosomes/sex chrom)			
	Number	Previously known	*New “de novo”*	Number	Previously known	*New “de novo”*	Number
Father	221	190/31	18/6	142	23/119	1/36	89
Mother	316	272/44	39/9	209	33/176	6/53	105
Daughter	352	261/92	34/25	421	68/353	5/108	106
Son **(patient)**	260	228/32	31/6	177	28/149	28/149	95

**Table 2:** Genes with de novo CNV alterations in the proband that are functionally associated to neurological phenotypes

GENE (OMIM)	Chromosome	Protein	Protein functions	Associated neuropathology
**CNV losses**				
*ATP1A2* (182340)	1q23.2	ATPase Na+/K+ Transporting Subunit Alpha 2	Na+/K+ ATPase maintains electrochemical gradient for electrical excitability of nerve and muscle. Participates in neurotransmitter uptake and muscle contraction [23, 24]	Rare forms of epilepsy and seizures [25–29]
*SLC17A5* (604322)	6q13	Solute Carrier Family 17 Member 5	Affects membrane potential-driven aspartate and glutamate transport into synaptic vesicles [30–32] Required for normal CNS myelination [33, 34].	Pathogenic variants or CNV loss of one allele associated to hypotonia, ataxia, epilepsy, seizures, nystagmus and findings of cerebral and cerebellar atrophy [33]
*ELAVL2* (601673)	9p21.3	ELAV Like RNA Binding Protein 2	Neural-specific RNA-binding protein that binds to several 3' UTRs. Expressed in early neuronal progenitors to mature neurons [35] and required for normal neuronal development in the embryonic CNS	Alterations in neuronal differentiation and regulates neurodevelopmental and synaptic gene networks [36]
*LRRC55* (615213)	11q21.32	Leucine Rich Repeat Containing 55	Subunit modulating gating properties in Ca^2+^- activated potassium channel BK, and its voltage dependence in the hyperpolarizing direction [37–40]	Altered neuronal polarization and depolarization [37–40]
*PCDH9* (603581)	13q21.32	Protocadherin 9	Ca^2+^-dependent cell adhesion in neural tissues. Protein involved in signaling at neuronal synaptic junctions [41, 42]	Epileptic encephalopathy [43]
*NALCN* (611549)	13q33.1	Sodium Leak Channel, Non-Selective	Voltage-gated Na^+^ and Ca^2+^ channels regulating the resting membrane potential and excitability of neurons [44–46]	*NALCN* deficiency is associated to channelopathies [47]. *NALCN* pathogenic variants associated to Neuroaxonal Dystrophy (INAD) patients, severe hypotonia, speech impairment, cognitive delay, epilepsy and mental disability [44, 45, 48, 49] *NALCN* associated to dystonia [46, 50]
*RYR3* (180903)	15q14	Ryanodine Receptor 3	Presynaptic endoplasmic reticulum ryanodine receptor-mediated Ca^2+^ release [51, 52]. Involved in skeletal muscle contraction by releasing calcium from the sarcoplasmic reticulum followed by depolarization of T-tubules Regulates composition of the protein complex that forms a voltage-independent, nonselective, non-inactivating cation channel permeable to Na^+^, K^+^, and Ca^2+^, which regulates the neuronal background sodium leak conductance [53]	*RYR3* haploinsuficiency cooperates *SCN1A*, implicated in epileps y[54, 55].
*CDH13* (601364)	16q23.3	Cadherin 13	Negative regulator of axon growth during neural differentiation [41, 43]	Epileptic encephalopathy [43]
*ANO3* (610110)	11p14.3	Anoctamine 3	Mutation in this gene exhibited abnormalities in endoplasmic reticulum-dependent calcium signaling, AbnormalCa^2+^-activated chloride channel [56, 57]	*ANO3* pathogenic variants have a dominant effect on dystonia [58, 59] and to complex neurological syndrome combining dystonia and myoclonus phenotypes [60, 61]
**CNV gains**				
*EFNA5* (601535)	5q21.3	Ephrin A5	Involved in short-range contact-mediated axonal guidance. Prevents axon bundling of cortical neurons with astrocytes [62]	
*PCDH19* (300460)	Xq22.1	Protocadherin 19	Calcium-dependent cell-adhesion protein primarily expressed in the developing brain [63, 64]	*PCDH19* pathogenic variants and CNV in epilepsy [63–69]

Next, we performed a search for a pathogenic association of all the genes comprised within these CNVs detected in the patient, by either SNP microarray or WES, and correlated their functions with different aspects of the clinical phenotype [17]. For this aim, the VarElect program was used [22]. All changes in the proband were also normalized with respect to the genome of the other family members, both parents and sister. The search was performed to identify functional and mechanistic correlations between gene functions and components of the clinical phenotype such as seizures, dystonia, epilepsy, neurotransmission, and motor neuron function. The neuropathogenicity of candidate genes within these genomic regions was determined by their previous association of their known mutations to a neurological phenotype. All the genes identified in the patient within CNVs that have a correlation with the clinical phenotype are expressed in neurons, have specific functions associated to the nervous system, or its known mutations have been associated to a neurological phenotype (Table 2). Functionally, most of these genes codify for several ion channel proteins or membrane proteins implicated in neuronal cell interactions, which can affect synaptic transmission and cell polarization. This indicates that individually these genes by themselves are not sufficient to cause the phenotype, but can contribute to the disease when they are combined with other genetic alterations in a unique individual. The affected boy presents a combined haploinsuficiency, mainly of CNV losses, that are likely to contribute to the pathogenic mechanism and the severity of the syndrome based on the expression level and subcellular localization of these proteins, the neurological functions associated to these proteins, and to the neurological pathogenic phenotypes associated to their genetic mutations in other patients.

**Fig. 1:**
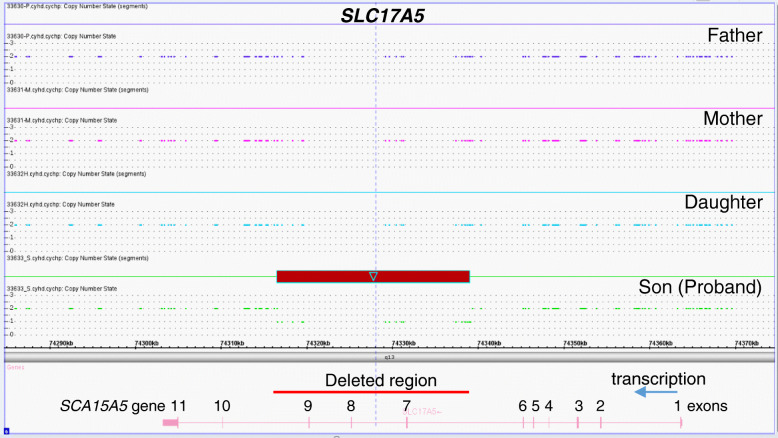
Exon deletion in *SLC17A5* gene in the proband. The genomic region comprising the *SLC17A5* gene in the four family members is shown. The region deleted in the proband is marked by a box and a red line. The arrow indicates the direction of transcription

One gene implicated in neurotransmission, *SLC17A5*, has a CNV loss that affects exons 7 to 9 (Table 3, Fig. 1). The SLC17A5 protein is required for the transport of aspartate and glutamate into synaptic vesicles, which are driven by the membrane potential [30]. However, it is not known whether a *SLC17A5* haploinsuficiency, expressing, and aberrant protein might behave in a manner similar to its mutants regarding symptoms such as dystonia, hypotonia, or seizure crisis and share some symptoms such as hypotonia, ataxia, epilepsy, nystagmus, and findings of cerebral and cerebellar atrophy detected in patients with Salla disease [33].

**Table 3:** Genes with exons altered by deletion or duplication

Gene	Exons deleted	Exons duplicated	CDS location of exons in gene sequence and aminoacids	NCBI Ref. sequence
*PCDH19*		3	59570-59628 (59 aa)	NG_021319.1
*SLC17A5*	7		32031-32189 (159 aa)	NG_008272.1
	8		38546-38678 (133 aa)	
	9		43446-43593 (148 aa)	
*RYR3*	2		162453-162572 (120 aa)	NG_047076.1
*ELAVL2*	3		95182-95285 (104 aa)	NG_016425.2
*ATP1A2*	2		5177-5281 (105 aa)	NG_008014.1
	3		5463-5522 (60 aa)	
	4		7484-7687 (204 aa)	
	5		8214-8327 (114 aa)	
	6		8567-8701 (135 aa)	
	7		9407-9524 (117 aa)	
*ANO3*	3		252796-252990 (195 aa)	NG_042856.1
	4		254643-254714 (72 aa)	
	5		273908-274026 (119 aa)

*RYR3* (ryanodine receptor type 3) codes for a presynaptic endoplasmic reticulum ryanodine receptor-mediated Ca2+ and forms a voltage-independent, nonselective, non-inactivating cation channel permeable to Na^+^, K^+^, and Ca^2+^, which regulates the neuronal background sodium leak conductance [70]. Functionally, RyRs proteins regulate the generation of plateau potentials in motor neurons and also affect vesicle mobilization and synaptic plasticity [70]. In motor neurons, the RYR3 protein regulates intracellular calcium, in which AMPA-type GluR (glutamate receptor) channels regulate the intracellular calcium homeostasis that is altered in neurodegenerative diseases and can play an important role in the pathogenesis of motor neuron disorders (MND) [71]. The patient has a CNV loss that includes exon 2 (Table 3, Supplementary Fig. S2). Furthermore, haploinsuficiency of *RYR3* might cooperate in an indirect way with several membrane proteins coded by genes implicated in sodium or calcium voltage channels, including *SCN1A* that is also implicated in epilepsy [54].

The NALCN protein is a sodium leak channel [72] expressed in neurons of the *substantia nigra*, and its reduction impairs the spontaneous firing required for the inhibition of downstream brain areas [73]. The NALCN protein interacts with UNC80 and pathogenic variants in both genes have been associated to dystonia [44, 74]. *NALCN* pathogenic variants have been associated to Neuroaxonal Dystrophy (INAD) patients, as well as to patients with severe hypotonia, speech impairment, cognitive delay, epilepsy, and mental disability [48]. The patient has a CNV loss, an intronic deletion in *NALCN* that alters its mRNA. Therefore, a reduction in the NALCN protein level might mimic a defective NALCN-UNC80 complex in the pathogenesis of dystonia. NALCN deficiency has been associated to channelopathies and cervical dystonia [47].

The SNP microarray analysis detected a large deletion that contains the *ANO3* (Anoctamine 3) gene coding for a protein belonging to the TMEM16 family that functions as a Ca (2+)-activated chloride channel. *ANO3* pathogenic variants have a dominant effect on dystonia [58], and some have been associated to a complex neurological syndrome combining dystonia and myoclonus phenotypes [60]. In the proband, *ANO3* has an LOH that incudes exons 3 to 5 (Table 3, Supplementary Fig. S3), and its combination with pathogenic variants and CVNs in other genes is likely to contribute to the syndrome.

Genes implicated in seizures and epileptic-like phenotypes present alterations in EEG patterns. The CNV analysis identified four genes with *de novo* alterations, three with loses (*ATP1A2*, *SLC17A5*, and *NALCN*) and one with a gain (*PCDH19*), which have a direct relation with epileptic-like phenotypes (Table 1). *ATP1A2* is highly expressed in the brain and codes for an integral membrane protein responsible for establishing and maintaining the electrochemical gradients of Na and K ions across the plasma membrane. *ATP1A2* has been associated, in several studies, to rare forms of epilepsy and seizures [25]. The proband has a loss comprising exons 2 to 7 (Table 3, Supplementary Fig. S4). Other genes with CNV losses that have an indirect relation to epilepsy are *RYR3*, *CDH13*, *PCDH9*, and *LRRC55* (Table 2).

Three of the affected genes in the proband, *PCDH19*, *PCDH9*, and *CDH13*, code for members of the cadherin protein family, which are implicated in neural intercellular interactions. *PCDH19* mediates cell adhesion in neural tissues and regulates signaling at synaptic junctions. Pathogenic *PCDH19* variants and CNV changes affect this gene in epilepsy [75]. More than one hundred different *PCDH19* pathogenic variants, located in the extracellular domain of the protein, have been associated with epilepsy, mostly in females, and recently were also detected in males [65]. Truncation mutations in *PCDH19* have also been associated to seizures [76]. *PCDH19* is located on chromosome X and the patient has a CNV duplication that includes exon 3 (Table 3, Supp. Fig. S5) that might alter its gene expression and protein stability or level and modify local interactions among neural cells. *CDH13* is a negative regulator of axon growth during neural differentiation [43], and a change in its protein level can also alter neuronal interactions and network organization. Both *CDH13* and *PCDH9* have an intronic deletion and a duplication, respectively, and both have been associated to epileptic encephalopathy [75]. Some of these cadherins are expressed in other organs such as the colon, kidney, heart, liver, and lung. However, the function of all these organs was normal in the proband.

Additional genes with CNV changes that can contribute to epileptic crisis and seizures were detected. *LRRC55* (Leucine Rich Repeat Containing or BK Channel Auxiliary Gamma Subunit) has a CNV loss deleting the 3′ untranslated region of the mRNA in the patient. *LRRC55* gene is expressed in the cortex, cerebellum, and spinal cord, and its protein is a regulator of large-conductance, voltage, and calcium-activated potassium channel (BK alpha) that modulates its gating properties [37]. Also there is a deletion of exon 3 in the *ELAVL2* gene (Supplementary Figure S6) coding for a neural-specific RNA-binding protein that binds to several 3′ UTRs and is expressed in early neuronal progenitors to mature neurons [35] and also regulates co-expression networks of neurodevelopmental and synaptic genes [36]. The *EFNA5* coding for ephrin5A also has an intronic duplication that can affect its transcription or RNA stability. Ephrin5 is implicated in neurodevelopment and axon bundling [62].

## Discussion

In patients with neurodevelopmental problems, there is a large genetic heterogeneity implicating several regions that present CNV changes. CNVs indicate a change in gene dosage, which pathogenically means that there is a lower, or higher, level of the protein in cells, depending on whether the type of change is a loss or a gain. Cellular functions are performed by proteins, and therefore, their levels and subcellular distribution are an important factor. The nervous system membrane-associated proteins, such as those that participate in ion and neurotransmitter channels, or in cell-cell interactions, are very likely to be affected depending of their level of expression and alteration in the number of functional complexes in the cell. Functionally, a change in protein levels is a mechanism different from mutant proteins. The change in protein level can affect its distribution and location in neuronal cell surface, leading to the alteration of complexes in which they participate. This is particularly relevant for membrane proteins that can affect cellular interactions, ion transport, or vesicle release, such as in synapsis. All these roles are essential for a normal neural development and its associated functions.

The clinical heterogeneity reflects a very complex situation in which an unknown combination of alterations in gene dosage and pathogenic variants is likely to contribute to the pathogenesis of complex neurological phenotypes. However, for a clinical phenotype, the cooperation of genes related to that particular phenotype are necessary, but the specific gene combination may vary from case to case and result in a neurological pathway perturbation that is pathogenic [77].

**Fig. 2:**
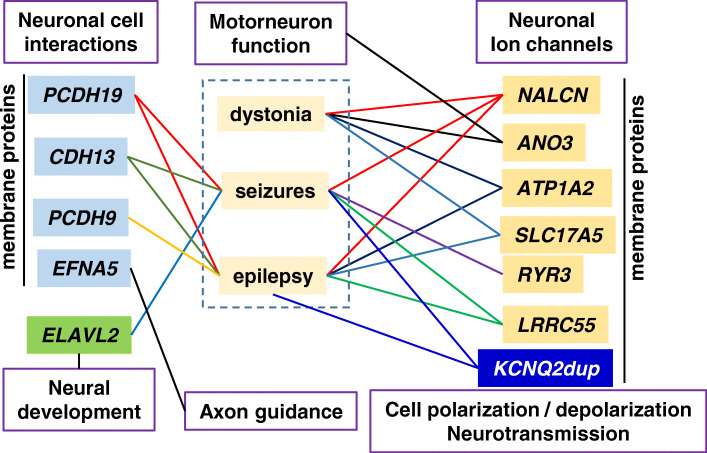
Pathogenic convergence between de novo altered genes and the clinical phenotype. The diagram illustrates the connection between OMIM genes with a pathogenic variant or CNV change that has a functional connection with the clinical phenotype of the patient. The lines indicate a direct association between a gene alteration (CNV or pathogenic variant) and the phenotype that has also been individually reported in other patients. The phenotypic associations were detected using the VarElect program to identify functional relations between genes and phenotypes. Dup: duplication

The contribution of individual genes with CNV changes to the patient phenotype is not known, but most of them occur in genes coding for proteins that participate in ion channels or neural cell interactions, which can regulate ion transport and neurotransmission. Thus, it is likely that an imbalance among several of these proteins and their functions contribute to complex neurological phenotypes. In the case of this patient, functions of the OMIM genes identified in the CNV analysis cluster by their mechanisms of action with the functional characteristics of the clinical phenotype (Fig. 2). The detection that several of the altered genes, directly or indirectly, are implicated in ionic (calcium, sodium) or glutamate transport and consequently are likely contributors to alterations in EEG, seizures, neurotransmission, epileptic-like crisis, or dystonia. Moreover, protocadherins and cadherin CNVs by altering their protein level and distribution on the cell surface can affect neural cell-cell interactions. Therefore, it is not possible to attribute a single phenotype to any individual gene, but the combination of several haplo-insufficient genes can generate a pathological situation related to their function. In this particular case, it is striking that four of the genes with a haploinsuficiency (*LRRC5*, *NALCN*, *RYR3*, and *ANO3*) affect calcium channels. CNVs in one of the implicated genes, *PCDH19* [75, 78], were already known to contribute to epileptic and neurodevelopmental delay syndromes [75].

The simultaneous alterations of several gene coding for different ion channels and cell-cell interaction proteins create a compound genetic haplo-insufficiency, which due to its complexity is likely to occur in patients presenting complex neurodevelopmental delays with an unknown etiology. What is relevant regarding the affected genes is their functional pathogenic combination rather than the implication of specific individual genes. It is important to consider that these neurodevelopmental phenotypes are likely to be the consequence of a complex pattern of alteration in gene dosage and expression levels of their proteins, rather that resulting from a unique monogenic defect, as it occurs with human variation in multiple phenotypes, normal or pathological. However, these complex clinical phenotypes can also be modulated by additional gene variants. It is likely that the clinical phenotype and its evolution will be conditioned by additional genetic alterations. Therefore, it is likely that alternative gene combinations can also cause related and similar clinical phenotypes.

Nowadays, the cause of cerebral palsy is changing from its origin in childbirth problems to an unknown origin, which is likely to be genetic and heterogeneous. It is possible that when unraveled, they might share common pathogenic pathways, although they might involve different genes that affect the same functions. The identification of gene/protein networks associated to the clinical neurological phenotype can set the bases for designing novel therapeutic approaches to manage these patients and minimize, or compensate, the functional consequences of neuropathogenic gene combinations.

## Conclusion

We conclude that the genetic heterogeneity of early severe neurodevelopmental delays, cerebral palsy-like with dystonia and epileptic encephalopathy, will have to be characterized in the context of the initiating pathogenic variant that is modulated by several additional genetic changes coding for proteins associated to neuronal functions.

## Supplementary Information

**Additional file 1: Supplementary Figure S1.** CNV from WES studies in the SLC17A5 gene. CNV alterations (Gains [red] and losses [blue]) retrieved by WES assays through the VarScan2 algorithm for the patient with respect to the other three family members. Graphs were depicted using the integrative genome viewer (IGV) tool for the GRCh37 human genome version.

**Additional file 2: Supplementary Figure S2.** Exon deletion in *RYR3* gene in the proband. The genomic region comprising the *RYR3* gene in the four family members is shown. The region deleted in the proband is marked by a box and a red line. The arrow indicates the direction of transcription.

**Additional file 3: Supplementary Figure S3.** Exon deletion in *ANO3* gene in the proband. The genomic region comprising the *ANO3* gene in the four family members is shown. The region deleted in the proband is marked by a box and a red line. The arrow indicates the direction of transcription.

**Additional file 4: Supplementary Figure S4.** Exon deletion in *ATP1A2* gene in the proband. The genomic region comprising the *ATP1A2* gene in the four family members is shown. The region deleted in the proband is marked by a box and a red line. The arrow indicates the direction of transcription.

**Additional file 5: Supplementary Figure S5.** Duplication encompassing exon 3 in the *PCDH19* gene. The genomic region comprising the *PCDH19* gene in the four family members is shown. The region deleted in the proband is marked bya box and a red line. The arrow indicates the direction of transcription.

**Additional file 6: Supplementary Figure S6.** Deletion encompassing exon 3 in the *ELAVL2* gene. The genomic region comprising the *ELAVL2* gene in the four family members is shown. The region deleted in the proband is marked by a box and a red line. The arrow indicates the direction of transcription.

**Additional file 7: Supplementary Table S1.** de novo CNVs (> 1Kb) identified in patient.

**Additional file 8: Supplementary Table S2.** Regions with loss of heterozygosity (LOH)> 3 Mb in the patient.

## Data Availability

SNP microarray data sets have been submitted to GEO with the identifier GSE122584. https://www.ncbi.nlm.nih.gov/geo/query/acc.cgi?acc=GSE122584 Whole Exome Sequence (WES) raw data files are available in: https://www.ncbi.nlm.nih.gov/sra/PRJNA629061 https://www.ncbi.nlm.nih.gov/bioproject/PRJNA629061 https://digital.csic.es/handle/10261/170405

## Abbreviations

PCDH19: Protocadherin 19
CDH13: Cadherin 13
PCDH9: Protocadherin 9
ELAVL2: ELAV-like neuron-specific RNA binding protein 2
NALCN: Na^+^ leak channel
ANO3: Ca^2+^-activated Cl^-^ channel
ATP1A2: ATPase Na^+^/K^+^ transporting subunit alpha 2
SLC17A5: Sodium-dependent Asp/Glu transporter
RYR3: Brain ryanodine receptor-calcium release channel
LRRC55: Auxiliary protein of the large-conductance, voltage, and Ca^2+^-activated K^+^ channel
EFNA5: Ephrin A5
