# Supplementary material for: Pathogenic convergence of CNVs in genes functionally associated to a severe neuromotor developmental delay syndrome
Source: Hum Genomics. 2021 Feb 8;15:11. doi: 10.1186/s40246-021-00309-4 (PMC7871650; doi:10.1186/s40246-021-00309-4)
Supplement: Supplementary file 1 — Additional file 1: Supplementary Figure S1. CNV from WES studies in the SLC17A5 gene. CNV alterations (Gains [red] and losses [blue]) retrieved by WES assays through the VarScan2 algorithm for the patient with respect to the other three family members. Graphs were depicted using the integrative genome viewer (IGV) tool for the GRCh37 human genome version. [file 40246_2021_309_MOESM1_ESM.pdf]

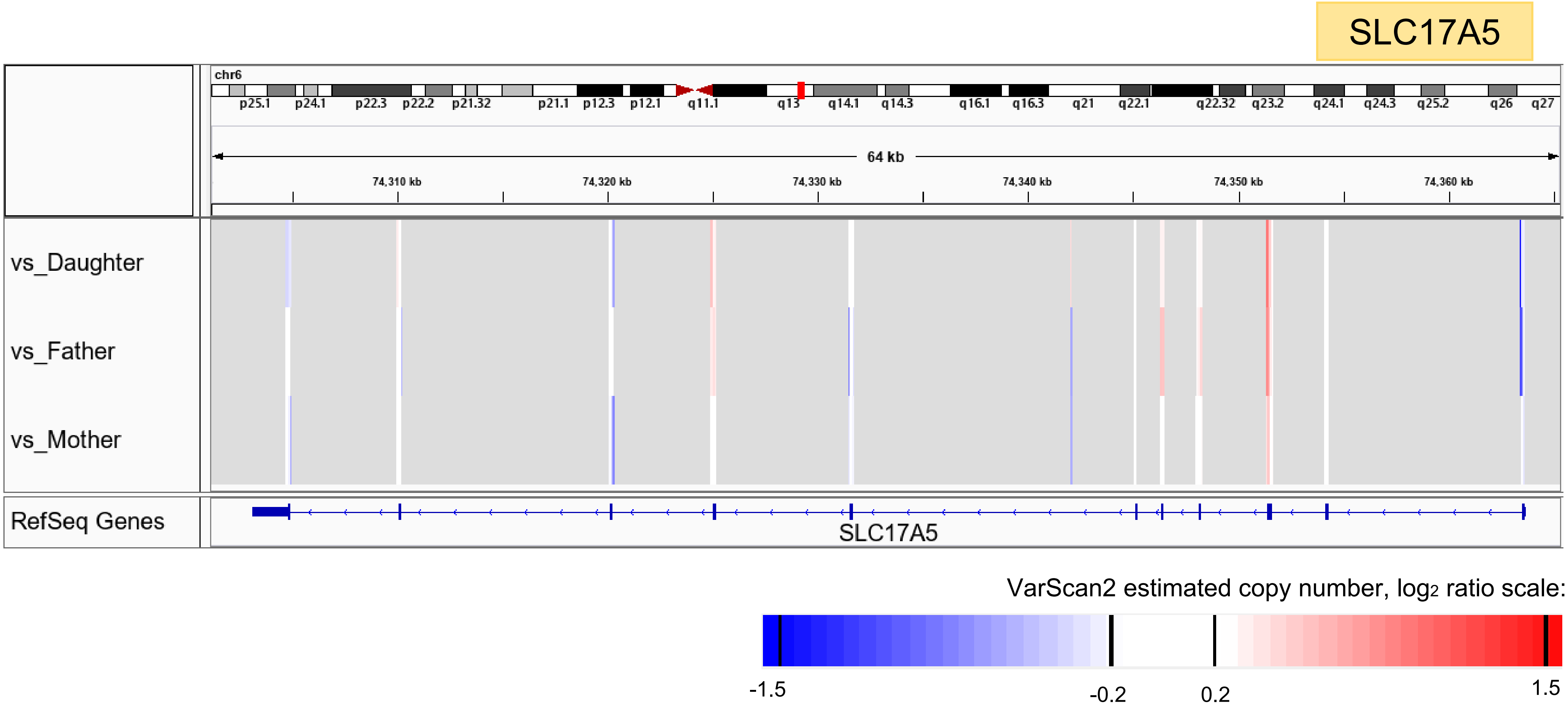

Supplementary Figure S1. CNV from WES studies in the SLC17A5 gene. CNV alterations (Gains [red] and losses [blue]) retrieved by WES assays through the VarScan2 algorithm for the patient with respect to the other three family members. Graphs were depicted using the integrative genome viewer (IGV) tool for the GRCh37 human genome version.
