# Supplementary material for: Pathogenic convergence of CNVs in genes functionally associated to a severe neuromotor developmental delay syndrome
Source: Hum Genomics. 2021 Feb 8;15:11. doi: 10.1186/s40246-021-00309-4 (PMC7871650; doi:10.1186/s40246-021-00309-4)
Supplement: Supplementary file 2 — Additional file 2: Supplementary Figure S2. Exon deletion in RYR3 gene in the proband. The genomic region comprising the RYR3 gene in the four family members is shown. The region deleted in the proband is marked by a box and a red line. The arrow indicates the direction of transcription. [file 40246_2021_309_MOESM2_ESM.pdf]

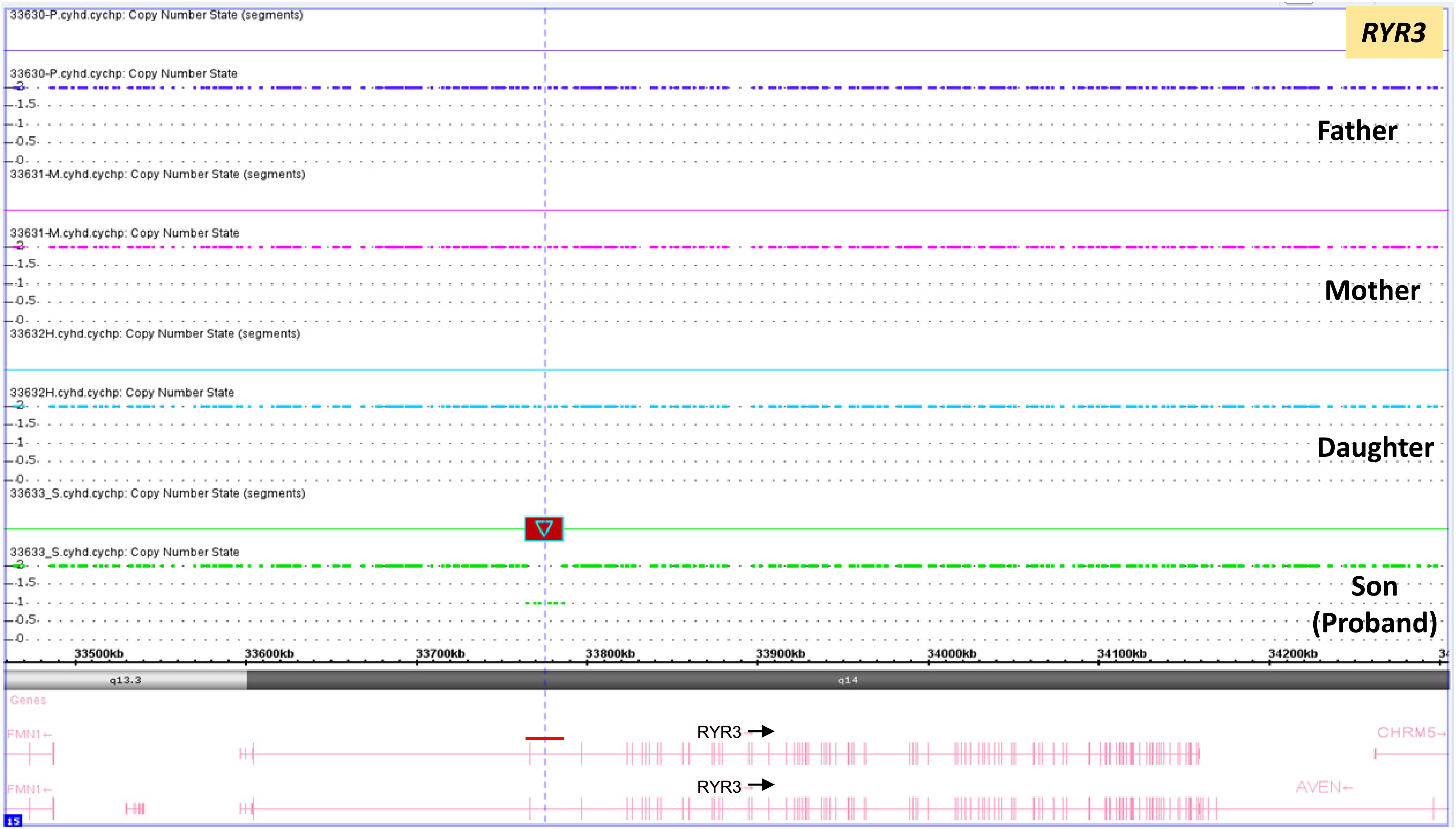

Supplementary Figure S2. Exon deletion in *RYR3* gene in the proband. The genomic region comprising the *RYR3* gene in the four family members is shown. The region deleted in the proband is marked by a box and a red line. The arrow indicates the direction of transcription.
