# Supplementary material for: Pathogenic convergence of CNVs in genes functionally associated to a severe neuromotor developmental delay syndrome
Source: Hum Genomics. 2021 Feb 8;15:11. doi: 10.1186/s40246-021-00309-4 (PMC7871650; doi:10.1186/s40246-021-00309-4)
Supplement: Supplementary file 5 — Additional file 5: Supplementary Figure S5. Duplication encompassing exon 3 in the PCDH19 gene. The genomic region comprising the PCDH19 gene in the four family members is shown. The region deleted in the proband is marked bya box and a red line. The arrow indicates the direction of transcription. [file 40246_2021_309_MOESM5_ESM.pdf]

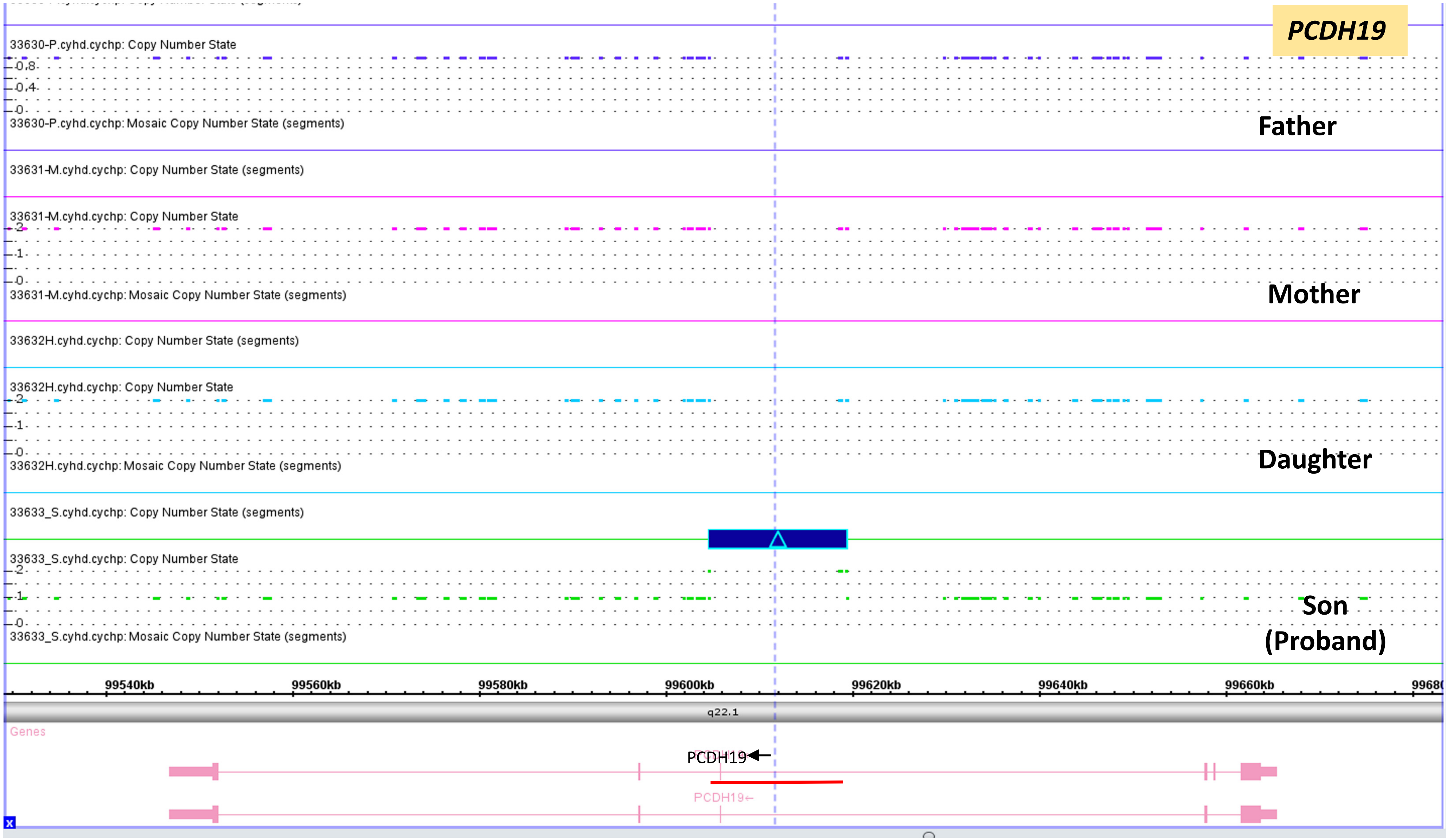

Supplementary Figure S5. Duplication encompassing exon 3 in the *PCDH19* gene. The genomic region comprising the *PCDH19* gene in the four family members is shown. The region deleted in the proband is marked by a box and a red line. The arrow indicates the direction of transcription.
