# Supplementary material for: Pathogenic convergence of CNVs in genes functionally associated to a severe neuromotor developmental delay syndrome
Source: Hum Genomics. 2021 Feb 8;15:11. doi: 10.1186/s40246-021-00309-4 (PMC7871650; doi:10.1186/s40246-021-00309-4)
Supplement: Supplementary file 6 — Additional file 6: Supplementary Figure S6. Deletion encompassing exon 3 in the ELAVL2 gene. The genomic region comprising the ELAVL2 gene in the four family members is shown. The region deleted in the proband is marked by a box and a red line. The arrow indicates the direction of transcription. [file 40246_2021_309_MOESM6_ESM.pdf]

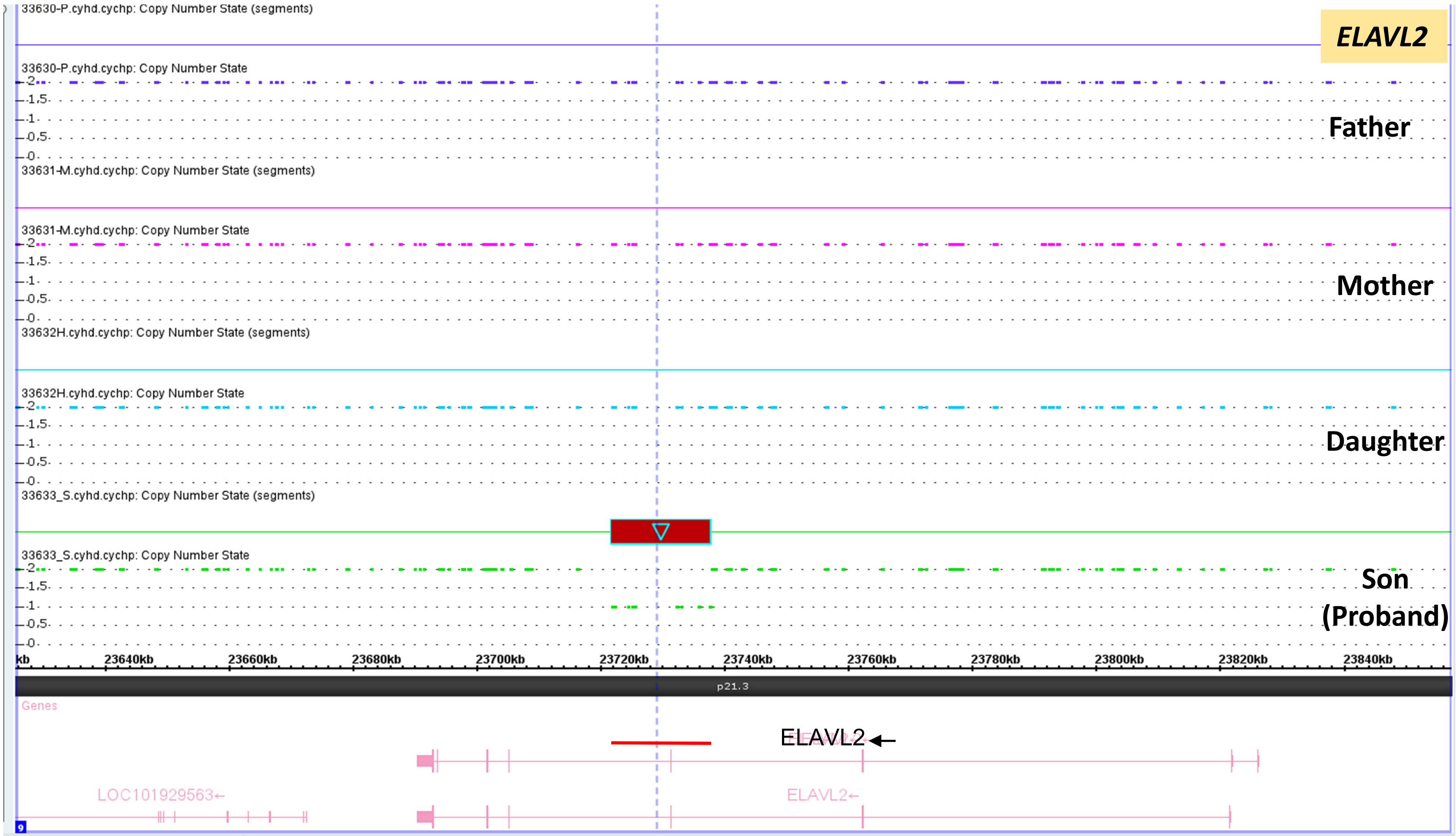

Supplementary Figure S6. Deletion encompassing exon 3 in the *ELAVL2* gene. The genomic region comprising the *ELAVL2* gene in the four family members is shown. The region deleted in the proband is marked by a box and a red line. The arrow indicates the direction of transcription.
