# Supplementary material for: Pathogenic convergence of CNVs in genes functionally associated to a severe neuromotor developmental delay syndrome
Source: Hum Genomics. 2021 Feb 8;15:11. doi: 10.1186/s40246-021-00309-4 (PMC7871650; doi:10.1186/s40246-021-00309-4)
Supplement: Supplementary file 7 — Additional file 7: Supplementary Table S1. de novo CNVs (> 1Kb) identified in patient. [file 40246_2021_309_MOESM7_ESM.pdf]

Supplementary Table S1

de novo CNVs (> 1Kb) identified in patient

Change	inherence	romoso	band S	toband E	Size (kbp	rker Co	OMIM ® Genes	Roles	Genes
GAIN	DE NOVO	5	q21.3	q21.3	6,557	5	**EFNA5 (601535)**	***axonal guidance***	EFNA5
	DE NOVO	13	q21.32	q21.32	13,662	36	***PCDH9 (603581)***	***indirect relation with epipleptic-like phenotype***	PCDH9
	DE NOVO	19	q13.42	q13.42	2,052	5			
	DE NOVO	22	q13.2	q13.2	1,363	5			
	DE NOVO	X	p11.22	p11.22	15,006	11			
	DE NOVO	X	p21.3	p21.3	3,214	12			
	DE NOVO	X	p21.3	p21.3	3,761	24	IL1RAPL1 (300206)		IL1RAPL1
	DE NOVO	X	p22.11	p22.11	7,63	24	SMS (300105)		SMS
	DE NOVO	X	q22.1	q22.1	14,739	7	***PCDH19 (300460)***	***direct relation with epipleptic-like phenotype***	PCDH19
	DE NOVO	X	q23	q23	24,223	61			
	DE NOVO	X	q24	q24	1,174	13			
	DE NOVO	X	q25	q25	6,256	9			
	DE NOVO	X	q25	q25	3,752	5			
	DE NOVO	X	q26.2	q26.2	1,204	16			
	DE NOVO	X	q26.3	q26.3	3,769	18			
	DE NOVO	X	q26.3	q26.3	1,039	12			
	DE NOVO	X	q28	q28	5,945	15			MAGEA9B, MAGE
	DE NOVO	X	q28	q28	1,241	12	CD99L2 (300846)		CD99L2
	DE NOVO	X	q28	q28	17,473	72	FLNA (300017)		FLNA
	DE NOVO	1	p13.1	p13.1	4,517	8	IGSF3 (603491)		IGSF3
	DE NOVO	1	p21.1	p21.1	16,579	12			
	DE NOVO	1	p22.2	p22.2	4,387	6	GBP1 (600411)		GBP1
	DE NOVO	1	p22.3	p22.3	2,638	8			

	DE NOVO	1	p31.1	p31.1	7,06	8	ST6GALNAC3 (610133)		ST6GALNAC3
	DE NOVO	1	p34.3	p34.3	26,157	12	OSCP1 (608854)		OSCP1
	DE NOVO	1	p36.32	p36.32	9,928	12			CEP104
	DE NOVO	1	q21.1	q21.1	70,518	51	NBPF9 (613999)		LOC100288142, NBPF9, N
	DE NOVO	1	q21.3	q21.3	22,781	12			
	DE NOVO	1	q23.2	q23.2	5,694	10	***ATP1A2 (182340)***	***direct relation with epipleptic-like phenotype***	ATP1A2
	DE NOVO	2	q12.3	q12.3	28,148	18	RANBP2 (601181)		RANBP2
	DE NOVO	3	p24.3	p24.3	12,303	7			
	DE NOVO	3	q11.2	q11.2	24,898	8			
	DE NOVO	3	q22.3	q22.3	12,185	12	PCCB (232050)		PCCB
	DE NOVO	3	q26.1	q26.1	7,653	8			
	DE NOVO	3	q27.1	q27.1	11,876	6			MCF2L2
	DE NOVO	3	q29	q29	34,906	12			SDHAP1
	DE NOVO	4	p15.33	p15.33	9,192	8			
	DE NOVO	4	p16.3	p16.3	3,039	5			FAM193A
	DE NOVO	4	q22.2	q22.2	10,03	8			
	DE NOVO	5	p13.3	p13.3	5,901	6			
	DE NOVO	5	p15.1	p15.1	3,668	13			
	DE NOVO	5	q11.2	q11.2	5,655	8	ANKRD55 (615189)		ANKRD55
	DE NOVO	5	q35.3	q35.3	80,37	76			ZNF354B, ZFP2
	DE NOVO	6	p12.3	p12.3	11,013	8	CRISP2 (187430)		CRISP2
	DE NOVO	6	q13	q13	22,495	20	***SLC17A5 (604322)***	***direct relation with epipleptic-like phenotype***	SLC17A5
	DE NOVO	6	q22.32	q22.32	7,794	8			
	DE NOVO	6	q23.2	q23.2	11,702	8	TBPL1 (605521)		TBPL1

LOSSES	DE NOVO	7	p14.3	p14.3	46,991	36	NPSR1-AS1 (608596), NPSR1 (608595)		NPSR1-AS1, NPS[illegible]
	DE NOVO	7	p15.3	p15.3	5,134	8	TRA2A (602718)		TRA2A
	DE NOVO	7	q11.21	q11.21	7,317	9	TPST1 (603125)		TPST1
	DE NOVO	7	q32.1	q32.1	11,833	12	TNPO3 (610032)		TNPO3
	DE NOVO	7	q34	q34	10,443	24	PRSS2 (601564)		PRSS3P2, PRSS2
	DE NOVO	8	p23.1	p23.1	2,463	6	MSRA (601250)		MSRA
	DE NOVO	8	p23.2	p23.2	7,925	12	CSMD1 (608397)		CSMD1
	DE NOVO	8	p23.3	p23.3	15,208	8			
	DE NOVO	8	q12.1	q12.1	12,412	12	NSMAF (603043)		NSMAF
	DE NOVO	8	q24.12	q24.12	9,326	6			
	DE NOVO	9	p21.3	p21.3	15,971	18	ELAVL2 (601673)		ELAVL2
	DE NOVO	9	q22.1	q22.1	7,098	8			
	DE NOVO	9	q34.13	q34.13	3,43	8	NUP214 (114350)		NUP214
	DE NOVO	10	p12.31	p12.31	6,292	12			
	DE NOVO	10	p12.31	p12.31	6,716	8			
	DE NOVO	10	p13	p13	3,167	12			
	DE NOVO	10	p13	p13	3,437	8			
	DE NOVO	10	p14	p14	9,561	11			
	DE NOVO	10	q23.1	q23.1	13,25	10			
	DE NOVO	11	q12.1	q12.1	11,36	10	***LRRC55 (615213)***	***indirect relation with epipleptic-like phenotype***	LRRC55
	DE NOVO	11	q14.3	q14.3	4,874	7			
	DE NOVO	11	q22.1	q22.1	11,109	8			
	DE NOVO	12	p12.2	p12.2	11,034	6			
	DE NOVO	12	q23.3	q23.3	9,476	8	RFX4 (603958)		LOC100287944, RF[illegible]
	DE NOVO	13	q13.3	q13.3	3,931	5			
	DE NOVO	13	q13.3	q13.3	38,162	18			

	DE NOVO	13	q21.32	q21.32	7,876	20	***PCDH9 (603581)***	**Epileptic encephalopathy**	PCDH9
	DE NOVO	13	q33.1	q33.1	3,389	8	***NALCN (611549)***	***direct relation with epipleptic-like phenotype***	NALCN
	DE NOVO	13	q33.1	q33.1	3,744	8	***NALCN (611549)***	***direct relation with epipleptic-like phenotype***	NALCN
	DE NOVO	13	q34	q34	7,663	8	COL4A2 (120090)		COL4A2
	DE NOVO	15	q11.2	q11.2	5,287	12	PWRN1 (611215)		PWRN1
	DE NOVO	15	q14	q14	21,858	20	***RYR3 (180903)***	**Dystonia**	RYR3
	DE NOVO	16	p12.3	p12.3	1,777	8	XYLT1 (608124)		XYLT1
	DE NOVO	16	p13.13	p13.13	7,687	12			
	DE NOVO	16	q23.3	q23.3	2,528	6	***CDH13 (601364)***	***indirect relation with epipleptic-like phenotype***	CDH13
	DE NOVO	18	q11.2	q11.2	19,583	10			
	DE NOVO	19	p13.12	p13.12	17,684	16			
	DE NOVO	22	q11.21	q11.21	8,033	10	SLC25A18 (609303)		SLC25A18
	DE NOVO	22	q13.31	q13.31	13,2	27	PPARA (170998)		PPARA
	DE NOVO	X	p22.33	p22.33	2,65	8	ASMTL (300162), ASMTL (400011)		ASMTL-AS1, ASM[illegible]
	DE NOVO	X	q23	q23	10,255	8	HTR2C (312861)		HTR2C
	DE NOVO	X	q24	q24	6,689	8			
	DE NOVO	X	q28	q28	5,02	16			arr[hg1[illegible]

Microarray Nomenclature
arr[hg19] 5q21.3(106,927,863-106,934,420)x4
arr[hg19] 13q21.32(67,272,417-67,286,079)x3
arr[hg19] 19q13.42(55,201,026-55,203,078)x4
arr[hg19] 22q13.2(41,072,903-41,074,266)x4
arr[hg19] Xp11.22(53,541,865-53,556,871)x2
arr[hg19] Xp21.3(25,074,293-25,077,507)x2
arr[hg19] Xp21.3(29,276,308-29,280,069)x2
arr[hg19] Xp22.11(22,004,718-22,012,348)x2
arr[hg19] Xq22.1(99,604,400-99,619,139)x2
arr[hg19] Xq23(115,133,383-115,157,606)x2
arr[hg19] Xq24(119,778,456-119,779,630)x2
arr[hg19] Xq25(122,230,315-122,236,571)x2
arr[hg19] Xq25(127,130,942-127,134,694)x3
arr[hg19] Xq26.2(133,173,923-133,175,127)x2
arr[hg19] Xq26.3(134,810,201-134,813,970)x2
arr[hg19] Xq26.3(135,532,754-135,533,793)x2
arr[hg19] Xq28(148,859,679-148,865,624)x3
arr[hg19] Xq28(149,968,359-149,969,600)x2
arr[hg19] Xq28(153,576,695-153,594,168)x2
arr[hg19] 1p13.1(117,119,919-117,124,436)x1
arr[hg19] 1p21.1(106,012,740-106,029,319)x1
arr[hg19] 1p22.2(89,523,245-89,527,632)x1
arr[hg19] 1p22.3(87,771,209-87,773,847)x1

arr[hg19] 1p31.1(76,917,405-76,924,465)x1

arr[hg19] 1p34.3(36,878,023-36,904,180)x1

arr[hg19] 1p36.32(3,744,260-3,754,188)x1

arr[hg19] 1q21.1(145,195,939-145,266,457)x1

arr[hg19] 1q21.3(154,333,126-154,355,907)x1

arr[hg19] 1q23.2(160,089,753-160,095,447)x1

arr[hg19] 2q12.3(109,337,737-109,365,885)x1

arr[hg19] 3p24.3(22,463,696-22,475,999)x1

arr[hg19] 3q11.2(95,454,290-95,479,188)x1

arr[hg19] 3q22.3(136,019,293-136,031,478)x1

arr[hg19] 3q26.1(162,314,772-162,322,425)x1

arr[hg19] 3q27.1(183,114,782-183,126,658)x1

arr[hg19] 3q29(195,690,227-195,725,133)x1

arr[hg19] 4p15.33(13,713,537-13,722,729)x1

arr[hg19] 4p16.3(2,681,413-2,684,452)x1

arr[hg19] 4q22.2(95,032,937-95,042,967)x1

arr[hg19] 5p13.3(32,987,711-32,993,612)x1

arr[hg19] 5p15.1(15,016,626-15,020,294)x1

arr[hg19] 5q11.2(55,426,360-55,432,015)x1

arr[hg19] 5q35.3(178,284,135-178,364,505)x1

arr[hg19] 6p12.3(49,668,364-49,679,377)x1

arr[hg19] 6q13(74,316,466-74,338,961)x1

arr[hg19] 6q22.32(126,675,340-126,683,134)x1

arr[hg19] 6q23.2(134,266,254-134,277,956)x1

arr[hg19] 7p14.3(34,673,682-34,720,673)x1

arr[hg19] 7p15.3(23,567,211-23,572,345)x1
arr[hg19] 7q11.21(65,733,326-65,740,643)x1
arr[hg19] 7q32.1(128,599,661-128,611,494)x1
arr[hg19] 7q34(142,475,398-142,485,841)x0
arr[hg19] 8p23.1(10,051,419-10,053,882)x1
arr[hg19] 8p23.2(3,468,179-3,476,104)x1
arr[hg19] 8p23.3(591,148-606,356)x1
arr[hg19] 8q12.1(59,511,848-59,524,260)x1
arr[hg19] 8q24.12(121,105,289-121,114,615)x1
arr[hg19] 9p21.3(23,721,531-23,737,502)x1
arr[hg19] 9q22.1(90,507,004-90,514,102)x1
arr[hg19] 9q34.13(134,066,956-134,070,386)x1
arr[hg19] 10p12.31(19,356,747-19,363,039)x1
arr[hg19] 10p12.31(19,679,317-19,686,033)x1
arr[hg19] 10p13(14,501,235-14,504,402)x1
arr[hg19] 10p13(16,015,462-16,018,899)x1
arr[hg19] 10p14(6,782,428-6,791,989)x1
arr[hg19] 10q23.1(86,601,979-86,615,229)x1
arr[hg19] 11q12.1(56,957,508-56,968,868)x1

arr[hg19] 11q14.3(90,328,742-90,333,616)x1
arr[hg19] 11q22.1(101,473,932-101,485,041)x1
arr[hg19] 12p12.2(20,064,688-20,075,722)x1
arr[hg19] 12q23.3(107,055,845-107,065,321)x1
arr[hg19] 13q13.3(37,957,087-37,961,018)x1
arr[hg19] 13q13.3(38,520,812-38,558,974)x1

arr[hg19] 13q21.32(67,501,784-67,509,660)x1

arr[hg19] 13q33.1(101,817,975-101,821,364)x1

arr[hg19] 13q33.1(101,893,499-101,897,243)x1

arr[hg19] 13q34(111,110,029-111,117,692)x1

arr[hg19] 15q11.2(24,805,897-24,811,184)x1

arr[hg19] 15q14(33,763,268-33,785,126)x1

arr[hg19] 16p12.3(17,479,996-17,481,773)x1

arr[hg19] 16p13.13(12,039,814-12,047,501)x1

arr[hg19] 16q23.3(82,679,436-82,681,964)x1

arr[hg19] 18q11.2(24,980,101-24,999,684)x1

arr[hg19] 19p13.12(15,033,591-15,051,275)x1

arr[hg19] 22q11.21(18,050,896-18,058,929)x1

arr[hg19] 22q13.31(46,601,910-46,615,110)x1

9] Xp22.33 or Yp11.32(1,520,755-1,523,405 or 1,470,755-1,473,405)x1

arr[hg19] Xq23(113,974,875-113,985,130)x0

arr[hg19] Xq24(119,960,837-119,967,526)x0

9] Xq28 or Yq12(154,941,868-154,946,888 or 59,044,874-59,049,894)x1
