# Supplementary material for: Pathogenic convergence of CNVs in genes functionally associated to a severe neuromotor developmental delay syndrome
Source: Hum Genomics. 2021 Feb 8;15:11. doi: 10.1186/s40246-021-00309-4 (PMC7871650; doi:10.1186/s40246-021-00309-4)
Supplement: Supplementary file 8 — Additional file 8: Supplementary Table S2. Regions with loss of heterozygosity (LOH)> 3 Mb in the patient. [file 40246_2021_309_MOESM8_ESM.pdf]

Supplementary Table S2. Regions with loss of heterozygosity (LOH)

Supplementary Table S2. Regions with loss of heterozygosity (LOH)> 3 Mb in the patient

Type	Chromosome	Cytoband Start	Cytoband End	CytoRegions	Size (kbp)	Marker Count	Gene Count	OMIM ® Genes Count

**LOH**	**16**	q22.1	q22.1	q22.1	3502,323	468	100	66

**LOH**	**16**	p11.2	p11.1	p11.2, p11.1	3590,042	236	18	1

Supplementary Table S2. Regions with loss of heterozygosity (LOH)< 3 Mb in the patient

Type	Chromosome	Cytoband Start	Cytoband End	CytoRegions	Size (kbp)	Marker Count	Gene Count	OMIM ® Genes Count
LOH	1	p36.13	p36.13	p36.13	1439,436	221	21	12

LOH	3	q22.2	q22.3	q22.3, q22.2	1404,567	229	7	6

LOH	3	q29	q29	q29	1102,963	177	12	8

LOH	4	p15.1	p15.1	p15.1	2121,445	508	0	0

LOH	4	q13.3	q13.3	q13.3	1262,461	276	5	5

LOH	5	q12.1	q12.1	q12.1	1014,239	235	5	3

LOH	5	q14.3	q14.3	q14.3	1199,182	298	3	1

LOH	5	q33.3	q33.3	q33.3	1257,535	296	17	11

LOH	7	q11.23	q11.23	q11.23	1323,292	49	23	8

LOH	7	q31.1	q31.1	q31.1	1162,935	292	3	2

LOH	8	q24.21	q24.21	q24.21	1100,735	295	1	0

LOH	9	q22.32	q22.33	q22.33, q22.32	1097,566	200	18	4

LOH	9	q34.11	q34.11	q34.11	1082,798	123	43	28

LOH	10	q11.22	q11.22	q11.22	1333,722	167	17	8

LOH	10	q26.13	q26.13	q26.13	1017,369	287	19	10

LOH	11	p14.3	p14.2	p14.2, p14.3	1165,542	521	1	1

LOH	12	q14.2	q14.3	q14.3, q14.2	1715,137	395	14	9

LOH	12	q24.31	q24.31	q24.31	1298,504	167	31	21

LOH	13	q21.31	q21.32	q21.32, q21.31	1211,013	300	0	0

LOH	13	q32.3	q32.3	q32.3	1326,902	364	13	6

LOH	19	p13.2	p13.2	p13.2	1030,625	219	31	11

OMIM ® Genes	Full Location

TRADD (603500), FBXL8 (609077), HSF4 (602438), NOL3 (605235), EXOC3L1 (614117), E2F4 (600659), ELMO3 (606422), MIR328 (613701), FHOD1 (606881), SLC9A5 (600477), PLEKHG4 (609526), TPPP3 (616957), HSD11B2 (614232), ATP6V0D1 (607028), AGRP (602311), CTCF (604167), CARMIL2 (610859), ACD (609377), PARD6A (607484), RANBP10 (614031), TSNAXIP1 (607720), CENPT (611510), THAP11 (609119), NUTF2 (605813), EDC4 (606030), PSKH1 (177015), CTRL (118888), PSMB10 (176847), LCAT (606967), SLC12A4 (604119), DPEP3 (609926), DPEP2 (609925), DDX28 (607618), DUS2 (609707), NFATC3 (602698), ESRP2 (612960), PLA2G15 (609362), SLC7A6 (605641), PRMT7 (610087), SMPD3 (605777), ZFP90 (609451), CDH3 (114021), CDH1 (192090), HAS3 (602428), CHTF8 (613202), UTP4 (607456), SNTB2 (600027), VPS4A (609982), COG8 (606979), TERF2 (602027), CYB5B (611964), NFAT5 (604708), NQO1 (125860), NOB1 (613586), WWP2 (602308), MIR140 (611894), CLEC18A (616571), CLEC18C (616573), EXOSC6 (606490), AARS (601065), DDX19B (605812), ST3GAL2 (607188), FUK (608675), COG4 (606976), SF3B3 (605592), IL34 (612081)	chr16:67174211-70676534

ZNF267 (604752) chr16:31630502-35220544

OMIM ® Genes	Full Location
NECAP2 (611624), NBPF1 (610501), MFAP2 (156790), ATP13A2 (610513), SDHB (185470), PADI2 (607935), PADI1 (607934), PADI3 (606755), PADI4 (605347), PADI6 (610363), RCC2 (609587), ARHGEF10L (612494)	chr1:16736737-18176173

PPP2R3A (604944), MSL2 (614802), PCCB (232050), STAG1 (604358), NCK1 (600508), IL20RB (605621)	chr3:135285824-136690391

XXYLT1 (614552), ACAP2 (607766), PPP1R2 (601792), APOD (107740), MIR570 (614538), MUC20 (610360), MUC4 (158372), TNK2 (606994)	chr3:194650488-195753451

chr4:32417743-34539188

MOB1B (609282), DCK (125450), SLC4A4 (603345), GC (139200), NPFFR2 (607449)	chr4:71709992-72972453

KIF2A (602591), DIMT1 (612499), IPO11 (610889)	chr5:61362329-62376568

COX7C (603774) chr5:84996731-86195913

SGCD (601411), TIMD4 (610096), HAVCR1 (606518), HAVCR2 (606652), MED7 (605045), ITK (186973), CYFIP2 (606323), NIPAL4 (609383), ADAM19 (603640), SOX30 (606698), CLINT1 (607265)	chr5:156038647-157296182

GTF2IRD1 (604318), GTF2I (601679), NCF1 (608512), GTF2IRD2 (608899), GTF2IRD2B (608900), TRIM73 (612549), TRIM74 (612550), HIP1 (601767)	chr7:73843868-75167160

IMMP2L (605977), DOCK4 (607679) chr7:110303526-111466461

chr8:129124948-130225683

SLC35D2 (609182), ZNF367 (610160), CDC14B (603505), CTSV (603308)	chr9:99066432-100163998

ENG (131195), AK1 (103000), ST6GALNAC6 (610135), ST6GALNAC4 (606378), PIP5KL1 (612865), DPM2 (603564), FAM102A (610891), NAIF1 (610673), SLC25A25 (608745), PTGES2 (608152), LCN2 (600181), CIZ1 (611420), DNM1 (602377), MIR199B (614791), GOLGA2 (602580), TRUB2 (610727), COQ4 (612898), SLC27A4 (604194), URM1 (612693), ODF2 (602015), GLE1 (603371), SPTAN1 (182810), WDR34 (613363), SET (600960), PKN3 (610714), ENDOG (600440), CCBL1 (600547), LRRC8A (608360)	chr9:130607711-131690509

RBP3 (180290), GDF2 (605120), GDF10 (601361), PTPN20B (610631), PTPN20A (610630), FRMPD2 (613323), MAPK8 (601158), ARHGAP22 (610585)	chr10:48365294-49699016

PLEKHA1 (607772), ARMS2 (611313), HTRA1 (602194), DMBT1 (601969), PSTK (611310), IKZF5 (606238), ACADSB (600301), HMX3 (613380), HMX2 (600647), BUB3 (603719)	chr10:124165614-125182983

ANO3 (610110) chr11:25377880-26543422

SRGAP1 (606523), XPOT (603180), TBK1 (604834), RASSF3 (607019), GNS (607664), TBC1D30 (615077), WIF1 (605186), LEMD3 (607844), MSRB3 (613719)	chr12:64434226-66149363

HCAR3 (606039), HCAR1 (606923), DENR (604550), CCDC62 (613481), HIP1R (605613), VPS37B (610037), ABCB9 (605453), ARL6IP4 (607668), PITPNM2 (608920), MPHOSPH9 (605501), C12orf65 (613541), CDK2AP1 (602198), SBNO1 (614274), SETD8 (607240), RILPL2 (614093), RILPL1 (614092), EIF2B1 (606686), GTF2H3 (601750), TCTN2 (613846), ATP6V0A2 (611716), DNAH10 (605884)	chr12:123201038-124499542

chr13:65352957-66563970

GPR18 (602042), GPR183 (605741), TM9SF2 (604678), CLYBL (609686), ZIC2 (603073), PCCA (232000)	chr13:99774561-101101463

PRKCSH (177060), ELAVL3 (603458), ZNF653 (611371), ECSIT (608388), CNN1 (600806), ACP5 (171640), ZNF627 (612248), ZNF69 (194543), ZNF20 (194557), ZNF136 (604078), ZNF44 (194542)	chr19:11509008-12539633

DGV	Segmental Duplications

nsv524363, nsv952044, nsv827707, nsv522852, nsv476845, nsv1160429, nsv524492, nsv471092, nsv457513, nsv572905, esv3582279, esv3638880, esv2079182, esv2714647, esv3553596, esv2657710, esv3638881, nsv572906, esv3553597, esv3638882, esv2668436, esv9318, nsv819753, nsv1144771, nsv827708, esv3638883, nsv833265, nsv1116155, esv3638884, nsv1841, esv3638885, nsv952045, nsv1142201, nsv833266, nsv827710, nsv1126431, esv2622215, esv2247849, esv3553599, esv2714648, esv2678491, nsv1070769, esv3638887, esv2659219, esv2714649, esv3638888, esv992883, esv3638889, esv29734, nsv827711, esv3329286, esv26764, nsv1070770, nsv1112693, esv3638890, esv2668512, esv3638891, esv2714650, nsv952046, nsv479975, nsv475320, nsv1842, nsv952047, esv2662518, esv3638893, nsv572907, nsv1127510, nsv1131266, dgv502e199, nsv1131268, nsv478420, nsv984342, nsv1131269, nsv1844, nsv977988, esv2591720, esv2670395, esv3638894, esv21655, nsv442402, esv3638895, nsv473126, nsv476694, esv2658552,	chr18:10722695, chrX:44492714, chr16:74433670, chr16:70200495, chr16:30230535, chr16:29491194, chr16:29388924, chr16:21409777, chr16:21841890, chr16:28463950, chr16:15453819, chr16:14843013, chr16:16428054, chr16:18408131, chr16:16471364, chr16:74408569, chr16:29046500, chr16:28350125, chr16:28763105, chr18:11615581, chr16:28635954, chr16:22547301, chr16:70233640, chr16:18448287, chr16:15195185, chr16:15029567, chr16:14803890, chr16:12018586, chr16:14781004, chr16:21416341, chr16:22511242, chr16:21848792, chr16:29395337, chr16:30236852, chr16:29496882, chr16:14800007, chr16:14838092, chr16:15473533, chr16:15502806, chr16:70239881, chr16:15507879, chr16:15059317, chr10:93874931, chr16:74559327, chr16:74477221, chr16:74365502, chr16:74362139, chr16:74425624, chr16:69977498, chr16:15453636, chr16:30230342, chr16:29491001, chr16:28463765, chr16:29063743, chr16:28783824, chr16:16487773, chr16:18407970, chr16:28669699, chr16:14859263, chr16:22547754, chr16:29388771, chr16:28349972, chr18:11615428, chr16:21841739, chr16:21409628, chr16:16444409, chr16:18448145, chr16:70006499, chr16:15045875, chr16:14820143, chr16:70036732, chr16:28637157, chr16:15481534, chr16:22496825, chr16:21444537, chr16:21876817, chr16:29423218, chr16:30265000, chr16:29525015, chr16:15507880, chr16:18996311, chr16:22552620, chr16:18867661, chr16:22474225, chr16:21896688, chr16:21464446, chr16:70404122, chr16:70365621

esv3570465, esv2714195, esv2658959, esv3638402, nsv833195, esv3892828, nsv571825, esv3638404, nsv1057598, nsv1786, nsv477931, esv3638406, nsv953805, nsv953806, nsv1078216, dgv50e203, esv3892829, esv3638408, esv3638410, esv3638409, esv3638411, nsv984311, nsv1787, nsv428325, nsv1108997, esv3638412, esv2678036, nsv472917, nsv833196, esv2640928, esv2375355, esv3638413, esv2666562, esv29641, nsv475846, nsv1113783, nsv977959, nsv9439, nsv833197, dgv2834n100, esv21568, nsv1160385, nsv1139654, esv3435168, nsv977960, nsv953807, nsv469789, dgv1517e59, nsv1070345, dgv2835n100, dgv2836n100, dgv2838n100, dgv2837n100, dgv2839n100, dgv2840n100, esv2662004, esv2714198, esv2714199, esv2714200, nsv1064477, nsv1145753, nsv977961, dgv2841n100, esv3638414, esv3638415, esv3892844, esv3892833, dgv4991n54, dgv2842n100, nsv433444, dgv814e212, dgv2843n100, dgv2844n100, dgv2845n100, dgv2846n100, dgv2847n100, dgv469n67, dgv815e212, dgv816e212, dgv817e212, nsv953808, dgv1518e59, nsv984312,	[illegible] chr16:31534502, chr16:31542908, chr16:33737687, chr16:32904579, chr14:106609994, chr17:36624863, chr17:36629191, chr16:33631903, chr16:32932087, chr17:36672410, chr14:106565208, chr17:25790936, chr17:25290286, chrUn_gl000217:151925, chr21:9439218, chr7:64601394, chr7:65016908, chr16:33573350, chr7:64971115, chr7:65112568, chr16:33853426, chr16:33817545, chr16:32854756, chr1:148851323, chr16:32819777, chr2:132792792, chr1:121138738, chr16:32127824, chr16:32289990, chr16:33230472, chr16:33287011, chr16:32659444, chr7:61807761, chr2:90400009, chr2:91621497, chr10:42642214, chr15:20104419, chr18:15194983, chr2:92254235, chr10:42608813, chr16:46462872, chr2:132767917, chr18:15165339, chr7:53217659, chr7:61727020, chr7:61072705, chr1:149009703, chr16:46490413, chr16:32143447, chr1:148669245, chr1:148831155, chrUn_gl000217:133304, chr15:20119266, chr21:9454093, chr16:33517468, chr7:57914379, chr16:33587509, chr7:64955704, chr16:32158141, chr16:32789357, chr13:19096310, chr21:14522769, chr7:61840744, chr1:149024090, chr7:61761635, chr7:61058196, chr7:61667275, chr16:46469279, chr10:42615237, chr7:53189339, chr7:64568472, chr9:70152742, chr9:69787720, chr9:42728303, chr7:65037865, chr2:91654391, chr9:70403673, chr2:92231783, chr2:91981968, chr2:90432584, chr16:33836119, chr17:25274445, chr18:15171691, chr2:132774259, chr16:32656338, chr16:34173150, chr7:64955765, chr18:15162965, chr21:10885038, chr10:42604348, chr16:46458853, chr2:92263073, chr2:132765315, chr7:57929149, chr10:42667608, chrY:13637319, chr10:38888849, chr22:16862531, chr1:121133097, chr15:20099636, chr1:148823084, chr16:32490080, chr10:38891096, chr16:32654335, chr7:65011431, chr16:33573232, chr7:65112510, chr16:33230604, chr16:33287143, chr16:32289989, chr16:32093043, chr7:61821678, chr10:42641894, chr16:32854257, chr16:33817547, chr2:91635751, chr2:90413825, chr7:64601390, chr17:25783496, chrUn_gl000217:122996, chr7:53223815, chr1:148661175, chr2:91616013, chr10:42652734, chr2:90394525, chr7:61727655, chr1:149004460, chr7:61803147, chr16:46474128, chr16:32103644, chr7:61062403, chr9:70165112, chr9:70416032, chr9:42719704, chr7:61360527, chr7:61659389, chr2:92244232, chr7:61833407, chr2:132808884, chr7:53202101, chr21:14511809, chr2:90425291, chr16:33828760, chr7:65050214, chr2:91974612, chr10:42619725, chr7:61767157, chr18:15210682, chr2:91647069, chr9:69800110, chr16:32837289, chr13:19085321, chr17:25263011, chr7:64567002, chr16:32110614, chr15:28458559, chr15:23310978,

DGV	Segmental Duplications
nsv829581, esv2675247, esv1035588, esv2674598, dgv19n67, esv2744408, esv2758925, nsv428421, dgv1n68, nsv9213, nsv9324, nsv436814, esv2750816, esv2677321, nsv159668, dgv10e201, nsv945793, dgv1e203, esv2678817, dgv46e59, esv33046, dgv47e59, nsv821511, dgv20n67, nsv829625, esv2744430, esv3367641, esv29642, nsv954846, dgv21e199, esv2744441, esv2670550, nsv955054, esv3385457, nsv472863, dgv48e59, dgv49e59, dgv50e59, nsv834203, nsv954847, esv3429389, nsv499577, nsv7173, nsv3632, nsv834247, nsv221, nsv471343, nsv482190, nsv471344, nsv945569, nsv957837, nsv514916, nsv945570, esv3419518, nsv435932, nsv159149, nsv436039, nsv945571, nsv945572, nsv545643, esv34697, esv3444386, nsv3743, dgv1n16, nsv954848, nsv160674, esv2744463, esv2597795, nsv469795, nsv471655, nsv3854, esv29948, dgv21n67, nsv436652, nsv945794, esv3385518, nsv945795, dgv51e59, nsv945573, nsv160333, esv6890, dgv158n54, dgv159n54, nsv545647, esv2674537, esv2744475, nsv957609, dgv2n6, dgv160n54, dgv161n54, dgv162n54, nsv545653,	chr1:147783151, chr1:149163313, chr1:16950916, chr1:17231952, chr1:21760557, chr1:17064394, chr1:16984774, chr1:149213428, chr1:147852602, chr1:143665090, chr1:149216600, chr1:17175658, chr1:147858380, chr1:143670863, chr1:149613619, chr1:144298080, chr1:144478309, chr1:144274481, chr1:149641238, chr1:143713812, chr1:144452606, chr1:147901321, chr1:149255634, chr1:17051487, chr1:16996058, chr1:149669362, chr1:149283809, chr1:149035883, chr1:144876700, chr1:21737596, chr1:146462471, chr1:145364801, chr1:21795116, chr1:148249825, chr1:148002506, chr1_gl000192_random:459805, chr1:146214650, chr1:146224432, chr1:145358539, chr1:146425855, chr1:146438580, chr1:146430620, chr1:146457711, chr1:144168168, chr1:144163123, chr1:146446365, chr1:148267938, chr1:148315659, chr1:144218734, chr1:148272712, chr1:144204421, chr1:144196466, chr1:148012600, chr1:148739635, chr1:148577550, chr1:144145783, chr1:144810724, chr1:145293306, chr1:148325179, chr1:146229449, chr1:146394748, chr1:149091770, chr1:146036159, chr1:147578085, chr1:16913464, chr1:146395242, chr1:16912274, chr1:120381503, chr1:148022852, chr1:149101094, chr1:144614531, chr1:16905978, chr1:149101083, chr1:148022846, chr1:16900901, chr1:144146732, chr1:144811667, chr1:148739139, chr1:148577053, chr1:21788085, chr1:149117254, chr1:21766304, chr1:120400041, chr1:146064160, chr1:147605996, chr1:148555349, chr1:144593043, chr1_gl000192_random:1270, chr1:146370380, chr1:148353247, chr1_gl000192_random:480590, chr1:145942148, chr1:147483860, chr1:147630262, chr1:146088425, chr1:17259346, chr1:16800937, chr1:21759439, chr3:49721419, chr3:18946795, chr1:17255338, chr1:16825367, chr1:147856951, chr1:143669434, chr1:17219869, chr1:16854740, chr1:17181559, chr1:149035881, chr1:21738004, chrX:20143899, chr1:6487720, chr1:144886480, chr1:17186107, chr1:16972126, chr1:149289470, chr1:149675011, chr1:143669425, chr1:147856942, chr1:234919133, chr1:146471298, chr1:144833969, chr1:145373855, chr1:149078766, chr1:16833539, chr1:144876721, chr1:149640537, chr1:143713108, chr1:149254933, chr1:147900617, chr1:149215313, chr1:16986202, chr1:147852605, chr1:143665093, chr1:16982973, chr1:17075728, chr1:16945458, chr1:21759410, chr1:246981308, chrX:99193218, chr11:62140541, chr6:56294447, chr3:126482969, chr4:109707285

nsv460860, nsv591829, esv2261145, esv2543430, nsv524122, esv2675945, esv29633, nsv515820, esv3363717, esv3305427, esv2666694, esv2662110, esv2660570, esv3448890, esv3306450, nsv829732, nsv473095, esv3369457, esv2671438, nsv4022, esv2669411, esv2421767, nsv4023, nsv4024, nsv967069, nsv955351, nsv965213, nsv508246, esv2760738, esv2665912, nsv237910, nsv4025, esv2725956, esv1521994, nsv821587, esv22097, esv9585, dgv8611n54, nsv591834, dgv8612n54, dgv37n50, esv2725957, dgv129n73, esv2662772, esv1003652, dgv8613n54, nsv591840, nsv514170, dgv8614n54, nsv591848, nsv591849, nsv520012, nsv238089, nsv591850, nsv591851, nsv460861, nsv4026, esv3368869, esv3306738, esv3327611, esv3410767, nsv955353, nsv979880, nsv472230, esv1010377, esv1391168, nsv965214, esv33758, esv3303749, esv3374061, esv33116, esv2676541, nsv4027, nsv507125, esv2725958, esv3397853, esv3419922	chr2:228733895, chrX:109764469, chr14:35898954, chr12:4668024

nsv519476, nsv469698, esv27232, esv2581881, esv3329293, esv3306340, nsv475576, esv2668033, nsv518682, nsv528661, esv2726452, esv2726453, esv2726454, nsv525763, esv2675794, esv2665937, nsv472029, esv1653494, esv2669079, nsv237463, esv2673526, esv27654, esv2726455, esv2726456, nsv592968, esv2726457, esv1432010, esv1454765, esv22519, esv24471, nsv511203, nsv476266, dgv8823n54, dgv8824n54, esv26277, dgv849n67, nsv819035, dgv904e199, dgv8825n54, dgv786e201, nsv950296, esv2726459, esv33213, nsv822407, dgv8826n54, nsv592978, nsv950297, nsv592981, nsv461126, nsv950298, esv3778, nsv592982, esv996963, esv2726461, esv2038835, nsv436791, esv3417334, esv2675653, esv3378262, nsv592983, nsv592984, esv28466, dgv8827n54, dgv8828n54, nsv592990, esv2666343, esv3324582, esv3304862, nsv526569, nsv963394, esv2759213, esv2726463, esv2669458, esv8403, nsv829824, esv2657233, nsv508977, esv2662107, nsv10379, nsv4181, esv2667844, nsv592991, nsv10380, nsv820668, esv27980,	chr3:195209724, chr3:195210104, chr3:195217533, chr3:195218320, chr3:195220442, chr3:195221533, chr3:195222910, chr3:195225635, chr3:195217496, chr3:195218891, chr3:195219192, chr3:195219495, chr3:195221952, chr3:195222240, chr3:195223025, chr3:195226233, chr3:195227407, chr3:195228420, chr3:195228900, chr3:195229457, chr3:195217725, chr3:195220143, chr3:195229763, chr3:195205891, chr3:195208824, chr3:195209728, chr3:195202319, chr3:195218511, chr3:195203772, chr3:195200000, chr3:195204146, chr3:195212913, chr3:195211637, chr3:195218549, chr3:195200001, chr3:195200002, chr3:195206423, chr3:195214894, chr3:195220660, chr3:195200003, chr3:195219119, chr21:37260056, chr2:107554856, chr6:32844254, chr5:156278142, chr3:195447355, chr3:195473005, chr3:197369994, chr5:1653485, chr5:1610340, chr5:266128, chr3:195682795, chr3:73113999, chr5:288235, chr3:897552, chr2:242968084, chr5:1594142, chr5:218030, chr5:1568461, chr5:261507, chr3:195341293, chr3:195347604, chr3:195511288, chr3:195510013, chr3:195506461, chr3:195505720, chr5:26217139, chr3:895257, chr8:70238816, chr3:195721776, chr5:270280, chr3:197359367, chr5:1612777, chr3:73113998, chr3:195367878, chr5:237611, chr3:197344663, chr5:26222121, chr3:195662287, chr5:26228056, chr2:242956216, chr2:26625, chr21:48096355, chr5:32649, chr2:164215

esv2759245, nsv593903, nsv442899, nsv524710, esv2668119, esv2653365, esv2345789, nsv593904, esv3356109, esv3306205, esv2464555, esv2334162, esv988525, esv2665114, dgv140n6, esv2727372, nsv956859, esv2236934, esv3389320, esv3303887, esv22715, nsv525305, nsv822521, nsv829897, esv2129909, esv2727373, esv2671781, nsv475008, nsv475899, nsv480712, nsv477907, esv8691, nsv956918, nsv956907, nsv525914, nsv481472, nsv479505, nsv472008, nsv479627, esv2431486, nsv4290, esv2671506, esv21883, nsv829898, nsv966259, nsv523344, esv29094, esv2030668, esv2727375, esv2668330, nsv4291, esv2677235, esv26095, esv275484, esv3307515, esv3408298, esv2727376, esv989795, dgv124e180, esv3301828, esv2671545, nsv520670, nsv470025, esv2533655, esv3374664, esv3442940, nsv593905, nsv291392, dgv83n17, esv3376271, nsv461321, nsv593906, nsv818226, nsv10476, esv2671379, esv22356, nsv517147, esv2422155, nsv442900, esv2760889, nsv514205, nsv509886, nsv4292, esv2727377, esv2659157, esv2727378,	chr8:136635813, chr20:31436477, chr7:57591796, chr20:26138079, chr7:57616300, chr20:26122674, chr17:20777777, chr17:21506101, chr17:22071425, chr7:57633140, chr17:20773705, chr20:26118541, chr17:21509996, chr20:26160439, chr7:57556950

nsv829966, nsv594651, nsv4381, esv2727796,
nsv594652, esv3325241, esv3333536,
esv2727797, esv2076165, esv4339,
nsv955684, esv1006939, esv1326491,
esv2727798, nsv441888, esv2762448,
esv25163, nsv516070, nsv822614,
esv2763808, esv3346423, nsv4382,
esv3354185, esv2727799, esv1975599,
nsv4383, esv2669073, nsv523289,
esv2476295, nsv950051, nsv4384, nsv525645,
nsv289841, nsv829967, nsv519903,
esv2677991, esv1487732, nsv822615,
nsv437395, nsv518154, esv3402603,
esv3304352, esv3427873, esv3309896,
esv3436214, esv1165662, esv2727800,
esv2185029, esv4331, esv2727801,
nsv955704, esv2727802, esv2727803,
nsv822616, esv2727804, esv2727805,
esv2727808, esv2727809, nsv955667,
esv7158, esv2033100, esv3924, esv2727810,
esv995866, dgv151n6, nsv955659,
esv1603902, esv3305821, esv3393306,
nsv508287, esv2289844, esv2663080,
nsv518185

nsv508363, nsv830317, esv2759345, nsv968180, nsv830318, nsv4857, esv2763457, nsv598322, dgv706n27, nsv471014, dgv9814n54, esv9730, nsv821303, esv22698, nsv10702, nsv964860, dgv3297e59, esv3337828, dgv3298e59, nsv964861, dgv3299e59, nsv598326, esv996027, esv23909, esv3440831, esv2665150, nsv598327, dgv9815n54, nsv823091, esv990716, dgv9816n54, dgv9817n54, dgv9818n54, nsv10703, nsv950071, nsv598348, esv35035, nsv968182, dgv1023e199, nsv10704, esv2662200, esv23269, nsv474336, esv3411385, nsv964862, esv2660465, nsv930480, esv2763458, nsv328503, esv3357380, nsv329525, esv2591326, esv2005948, nsv328501, dgv941e201, dgv942e201, nsv955846, nsv4858, nsv4859, esv2730255, esv2730256, esv2759346, nsv10706, esv34525, nsv964863, nsv521534, dgv948n67, esv2730257, esv2673244, esv2730258, nsv327506, nsv830319, esv2671688, esv2760964, nsv950072	chr16:33426009, chr15:20390970, chr15:21397632, chrX:146036557, chr1:78781859, chr10:29984844, chr2:85189962, chr9:88879467, chr2:129274864, chr17:52555161, chr19:19937825, chr19:21562530

nsv462240, nsv598822, esv2422510, esv1409944, nsv329250, nsv327614, dgv9917n54, dgv9918n54, esv2676756, nsv507271, esv2762529, nsv509975, esv1617263, nsv598833, esv2730416, nsv598834, esv1944398, esv2665313, esv2730418, esv2669543, nsv483007, nsv10713, esv2730419, esv1952008, esv2730420, nsv327400, esv1095730, nsv10714, esv26737, nsv955708, esv2675188, nsv968960, nsv968961, dgv9919n54, nsv462245, nsv598838, nsv818355, nsv509976, esv1001640, nsv327420, esv1390094, esv3428885, esv3304277, esv2563889, dgv201n6, esv3359009, esv3305770, esv3400686, nsv830375, esv2730421, nsv598839, nsv830376, esv3395633, esv2658577, nsv474611, nsv477365, nsv598840, nsv830377, nsv598841, nsv511303, dgv952n67, nsv820329, esv5749, esv24059, esv991086, dgv148e180, nsv513246, esv2730422, esv2666610, nsv598842, dgv9920n54, dgv9921n54, dgv9922n54, nsv442953, dgv9923n54, nsv514313, dgv9924n54, nsv598867, dgv9925n54, nsv598869, nsv598874, esv2730423	chr1:108900941, chr1:108989309, chr1:108755625, chr3:120043417

nsv830523, nsv830524, esv2657305, esv6048, nsv518243, dgv358n21, nsv522295, nsv969006, esv2730953, esv2674952, esv1009526, esv1004108, esv2667486, esv2730954, nsv477873, nsv474311, nsv528418, esv2661490, esv2730955, nsv515686, nsv950432, nsv474929, nsv830526, nsv600132, esv2730956, esv2678778, nsv968264, esv3387468, nsv5089, nsv600133, nsv462497, esv997268, esv6935, esv2759389, esv2730957, esv2730958, esv2730959, esv2548260, nsv980747, nsv527582, esv2730960, nsv600134, nsv10766, nsv474809, nsv473743, nsv5090, nsv509097, esv1006771, nsv521000, nsv479605, nsv471941, esv22643, esv2674272	chr21:37259295, chr6:32844249, chr13:64384484, chr2:107554853, chr3:195241225, chr19:24341423, chr5:157116659, chr22:31051281, chr5:157077834

nsv831032, nsv5793, nsv831033, esv3363878, nsv476326, nsv981527, esv2669330, esv33268, nsv966848, esv3414828, nsv517573, nsv5794, nsv966849, nsv971395, nsv8141, nsv966851, nsv970532, nsv509208, nsv8142, nsv482102, nsv7402, esv3352039, nsv8143, esv2761338, nsv971396, nsv981528, esv24294, nsv482104, nsv8144, nsv981665, nsv510089, nsv971037, esv3416980, nsv950551, nsv981666, nsv970533, nsv981529, nsv8145, nsv950552, esv2658348, nsv970534, nsv970882, nsv981530, nsv951355, esv1398337, nsv607486, esv3287828, esv23295, nsv820906, dgv11446n54, nsv607489, dgv11447n54, nsv607491, dgv11448n54, dgv11449n54, nsv607498, nsv528969, nsv970883, esv3341746, nsv831034, nsv8146, nsv607499, nsv8148, nsv8149, nsv970884, nsv967334, nsv970885, nsv970535, nsv951356, nsv8150, nsv971397, nsv8151, nsv981531, nsv820756, esv28657, nsv981532, nsv970536, esv3379624, esv3444656, nsv971038, nsv981667, esv3426613, esv3441281, esv23669, nsv607500, nsv607501, nsv821338, dgv11450n54, dgv11451n54, dgv11452n54, nsv981533, nsv981668, nsv970537,	chr7:66303060, chr7:75801485, chr7:66279357, chr7:65976158, chr7:66301034, chr7:65978173, chr7:65982168, chr7:65993397, chr7:66000101, chr7:66006098, chr7:72588834, chr7:74489986, chr7:72455742, chr7:74959150, chr7:72819983, chr7:72694592, chr7:99793993, chr7:99911392, chr7:99779749, chr7:102019334, chr7:6040350, chr7:66745441, chr7:76666548, chr7:72505218, chr7:74908585, chr7:74931125, chr7:101975581, chr7:74694336, chr7:75125369, chr7:76150897, chr7:76640837, chr7:101976995, chr7:6748938, chr7:5054531, chr7:44041274, chr7:102294507, chr7:102195407, chr7:72329132, chr7:102290855, chr7:102191762, chr7:44037585, chr7:76687870, chr7:101994521, chr7:66740071, chr7:72340252, chr7:76168818, chr7:99910002, chr7:99908612, chr7:99895161, chr7:75119840, chr17:57258595, chr7:74765724, chr7:72531393, chr7:99881480, chr7:74633605, chr7:99870374, chr7:99819660, chr7:74142511, chr7:72506861, chr7:99854072, chr7:74340922, chr7:74847765, chr7:74963942, chr7:72478889, chr7:74936723, chr7:99903894, chr7:74309156, chr7:75121878, chr7:66742207, chr7:44038075, chr7:102192252, chr7:102291345, chr7:76668180, chr7:44041418, chr7:75125374, chr7:74340888, chr7:99903081, chr7:74307861, chr17:57248011, chr7:101975687, chr7:74959099, chr7:99898320, chr7:74694325, chr7:74931074, chr7:74286049, chr7:74694102, chr7:72813080, chr7:72786996, chr7:72700442, chr7:76253689, chr7:101977100, chr7:5075536, chr7:75144446, chr7:6759941, chr7:66738131, chr7:76658069, chr7:72496683, chr7:74327547, chr7:99901954, chr7:74934780, chr7:74962805, chr17:57247875, chr7:44037582, chr7:102290852, chr7:102191759, chr7:76668708, chr7:74694564, chr7:72524745, chr7:74908813, chr7:99909993, chr7:72507381, chr7:74309673, chr7:72479405, chr7:74696575, chr7:5059905, chr7:76279229, chr7:72349310, chr7:75103388, chr7:102003633, chr7:5084888, chr7:6768803, chr7:76178033

nsv608080, nsv608147, nsv464671, dgv11570n54, esv2661784, nsv478799, nsv464673, nsv608150, esv2671616, nsv608151, esv3431116, esv2674667, esv21685, esv2421473, esv2761159, nsv442061, nsv514439, esv2672432, esv2735023, esv3354493, esv3309484, esv26791, esv3303910, esv3324264, nsv365322, dgv11571n54, esv3389118, nsv526883, esv2759554, nsv476818, esv2385229, esv5171, esv2735024, dgv235n6, nsv958411, esv1010951, esv8850, esv3906, esv2735025, esv2659693, esv989131, esv1139378, nsv955977, esv9669, esv2735026, nsv11, nsv5898, esv2657607, nsv475901, nsv475701, esv2673039, nsv608154, nsv824267, nsv608155, nsv515693, esv3417036, nsv608156, nsv464674, esv2752141, nsv428183, esv2759555, nsv464675, nsv608157, nsv470384, dgv427n21, nsv437027, esv26741, esv2762694, nsv519432, nsv818545, esv2661413, dgv29e196, dgv428n21, esv6655, esv3377857, esv1003541, esv1751628, esv3432287, esv2735030, nsv437998, dgv11572n54, esv2667081, esv2663569, esv2599634, nsv608160, esv2735031,	chr12:23037143, chr4:144730099

nsv7419, nsv499795, nsv6388, esv2657611, nsv528321, nsv507492, esv275073, esv2737569, nsv520611, esv2737570, nsv6389, esv2737571, esv3430899, esv2737572, esv3385385, esv2737574, nsv971352, nsv6390, nsv476782, nsv6391, esv275241, nsv508527, esv2761232, esv2635480, esv9266, esv2019272, nsv512067, dgv190e180, nsv612172, nsv465806, esv3520, nsv957670, esv3270274, nsv499732, dgv255n6, esv2737575, esv1765110, nsv436577, esv2676069, nsv465807, nsv612173, nsv527164, esv274913, esv2759641, esv28916, nsv475173, nsv480777, esv3410295, esv3308758, esv3307528, esv3390775, esv1219720, esv3394157, nsv509277, nsv6392, esv3310429, esv3399749, nsv476822, nsv508528, esv2764064, dgv12310n54, esv2659259, dgv12311n54, esv22071, esv2667830, nsv499338, esv988890, nsv820809, dgv12312n54, dgv12313n54, nsv612189, dgv12314n54, dgv12315n54, dgv12316n54, esv2421643, nsv612199, nsv612204, dgv12317n54, nsv6393, nsv473379, esv2578193, nsv951469, dgv12318n54, esv2657497, esv23187, [illegible]	chr12:14369515, chr11:114217609, chr20:62904852

nsv824998, nsv968736, esv3328456, esv3429931, nsv416131, dgv12803n54, nsv472681, nsv614924, esv22781, esv2669547, dgv12804n54, nsv614930, nsv614933, nsv950906, nsv968737, nsv522838, nsv982325, esv2306628, esv2089533, esv6348, nsv415660, nsv824999, esv2442788, esv1923447, dgv1360e199, esv2673087, esv2761546, nsv950907, nsv416110, nsv972428, nsv831660, nsv520001, nsv972429, nsv6624, esv3305699, esv3323096, nsv950908, nsv475931, esv2668332, nsv831661, nsv473484, esv2738813, esv2663765, esv2535600, esv2661849, nsv442560, nsv982326, nsv972430, nsv972431, nsv972788, nsv950909, esv28215, nsv972789, nsv6625, nsv831662, nsv972790, nsv950910, esv25444, nsv6626, nsv972432, nsv973382, esv2675029, esv2668826, esv2670739, nsv969695, nsv415491, nsv982327, nsv972433, esv3310505, esv3327131, nsv972791, esv1727048, esv2762823, esv2761547, esv3327906, esv3305119	chr7:48881751, chr5:53041016, chr10:103934641, chr7:48965665, chr12:88176700, chr1:43356587, chr9:97069068, chr10:89115603, chr10:81599292, chr10:81462769, chr10:88984991, chr10:89117211, chr10:81600900, chr10:52436248, chr9:97122780, chr9:38475368, chr7:6369046, chr9:38543969, chr9:33511239, chr9:84670187, chr9:90539277, chr9:90730601, chr9:95650715, chr1:143200000, chr3:197782986, chr8:43105721, chr9:95570617, chr6:26634615, chr2:133110282, chrY:13262550, chrUn_gl000243:29533, chrUn_gl000214:108559, chr21:9708049, chr4:49272573, chr1:143270154, chr4:49525503, chr9:45355990, chr9:66493143, chr9:45352101, chr9:66489234, chr13:41455741, chr13:52744208, chr9:33509234, chr9:33484738, chr13:20103469, chr4:85387050

nsv615374, nsv466575, nsv466576, dgv12880n54, nsv615377, nsv615378, esv28616, nsv615379, dgv1377e199, esv2739054, dgv497n21, nsv615380, nsv615381, nsv466577, esv2739055, nsv6722, nsv831727, nsv478648, esv2739056, esv5200, esv2739057, nsv476947, nsv951768, esv2671313, nsv825096, esv2660141, nsv471324, esv2670778, esv26069, nsv520376, nsv615382, esv28697, nsv518112, nsv615383, nsv818724, nsv951769, nsv6723, esv2739058, esv2739059, esv2778, esv1157058, esv2678543, nsv951770, esv1009770, nsv982346, esv2739060, dgv1265e201, esv2743007, dgv1266e201, nsv951771, esv1129526, esv2739065, nsv512118, esv2901, esv1706831, esv8629, esv2739067, esv2739068, esv1725840, nsv951772, nsv831728, nsv972807, nsv951773, nsv972808, esv2739069, nsv972453, nsv415888, esv2739070, esv2660941, nsv951774, dgv1378e199, esv2739071, nsv956125, esv1113963, dgv1379e199, esv2739072, esv2150257, nsv956099, esv1639805, esv2618000, nsv526979, esv3423649, esv2739074, nsv416781, esv2666546, nsv615384,	chr15:78198639, chr12:107665276, chr14:51904337

esv2759747, nsv510991, nsv8636, nsv436102, nsv7203, esv2736163, nsv550763, nsv467059, esv21870, nsv831860, nsv831861, nsv528799, esv2678415, esv2672759, esv2670465, esv3398674, esv3306598, esv3309440, esv3425650, esv1081992, esv1010922, nsv476735, esv6330, nsv475156, esv2674312, esv3302822, esv995742, esv1308340, nsv957742, esv2470698, esv2459256, esv2008130, esv2661026, nsv442179, esv2421380, esv2759748, nsv467189, nsv471969, esv33233, dgv136e199, nsv951589, nsv947966, nsv820767, nsv831862, nsv951590, nsv550803, nsv433259, nsv6554, nsv550804, nsv947967, dgv137e199, nsv498727, nsv971752, nsv974941, nsv982761, nsv971755, nsv550805, nsv947968, nsv947969, nsv469543, nsv947970, nsv550806, nsv947971, nsv947972, nsv469669, esv24863, nsv820931, nsv947973, nsv550807, esv33214, nsv947974, dgv12e203, nsv437672, nsv982763, nsv831863, nsv975693, nsv975696, nsv947975, dgv1121n54, nsv550810, nsv831864, nsv950951, nsv8637, nsv8638, nsv6565, nsv8639, nsv550811, nsv467190, esv1001778, nsv6576, nsv550812,	chr10:46476964, chr10:49268060, chr10:57569494, chr10:47529169, chr10:81390559, chr10:58185253, chr10:46843511, chr10:47324316, chr10:49033267, chr10:51448845, chr10:81675581, chr10:47576087, chr10:88787402, chr10:75497650, chr10:88760721, chr10:46171743, chr10:47703925, chr10:46319300, chr10:51732600, chr10:51222928, chr10:75432278, chr10:48105707, chr10:47189999, chr10:49216137, chr9:76262564, chr10:38456658, chr10:42956662, chr10:42960773, chr10:43276748, chr10:88751887, chr10:81663255, chr10:52498045, chr10:88828344, chr11:1576025, chr10:52467445, chr10:81632438, chr9:90456215, chr10:89148741, chr10:56028403, chr10:56177753, chr10:57346176, chr10:81390560, chr10:46692890, chr10:48879443, chr10:58190503, chr10:81672210, chr10:47576096, chr10:88870572, chr14:88418873, chr10:47767474, chr10:48275802, chr10:47152145, chr10:47746316, chr10:47171126, chr10:48187336, chr10:88760724, chr10:51211554, chr10:46307923, chr10:75419335, chr10:51462405, chr10:46712815, chr10:48899348, chr9:76262565, chr10:42956751, chr10:43279736, chr10:88752022, chr10:81666346, chr10:48747514, chr10:46560617

nsv825594, nsv7580, esv9290, esv28769, dgv1409n54, esv2663177, nsv552253, nsv552254, nsv955802, nsv516138, nsv8729, nsv428240, esv2759786, nsv552255, esv23096, nsv508611, nsv8730, nsv7581, esv2760157, esv2764158, nsv55, nsv7582, nsv825595, nsv948209, nsv8731, nsv819547, nsv499308, nsv825596, nsv436043, nsv442592, esv2422149, esv2658567, nsv951865, nsv820718, dgv1410n54, dgv1411n54, dgv175n67, dgv176n67, esv33340, nsv514541, dgv1412n54, nsv971857, nsv948210, nsv7583, nsv507579, nsv56, nsv821496, esv1000689, nsv951866, nsv499097, dgv1413n54, nsv552275, esv2676690, nsv948211, nsv514542, nsv24315, esv2362486, esv22906, nsv7584, esv3361994, nsv509370, esv3308621, esv3306648, esv3340044, nsv7585, dgv516e59, nsv25104, nsv478272, esv3397858, esv3306380, esv3421978, nsv24903, nsv832010, nsv975804, esv7958, esv6242, esv2453822, esv3305325, esv3309892, esv1608173, esv3441593, nsv7586, esv2674518, nsv474515, nsv948213, nsv476827, nsv7587, esv3376979, esv3304322, esv3422703, nsv958565,	chr10:124331382, chr10:124329954

nsv553827, esv2422339, nsv467763, nsv553860, esv2760599, nsv553864, nsv832090, esv3440336, nsv553867, nsv521288, nsv523452, nsv442224, nsv521676, esv2744251, esv2321478, esv2744254, nsv38658, esv988937, nsv957220, esv2744255, esv1982398, esv3370, esv2744256, esv2760164, nsv951322, esv2744257, esv2744258, esv2751021, esv3446977, esv2644038, nsv467764, dgv1718n54, dgv196e199, esv2759811, esv2744259, esv4856, esv2661825, esv3258163, nsv437708, nsv553870, nsv467765, nsv553871, nsv553872, esv3380680, esv1061768, nsv437709, esv2760212, nsv8799, esv2678072, esv26376, nsv982997, esv2421640, nsv442606, nsv514604, esv2667377, nsv517418, nsv818806, esv1136147, esv1261238, nsv428252, nsv520281, nsv526726, dgv1719n54, nsv8800, esv2520553, nsv526614, esv2744260, esv2744261, esv2164088, esv2744262, esv1005260, nsv38780, esv1278199, nsv38539, nsv553876, nsv553877, nsv7716, esv2760183, dgv136n27, dgv1720n54, dgv33n17, nsv553880, esv2647190,	chr1:227817058, chr12:7335127

nsv832442, esv33314, esv2676422, esv2745986, esv1536875, nsv469456, nsv559202, nsv517246, esv1457683, esv2745987, nsv958682, esv2745988, nsv472434, nsv832443, esv2745989, nsv832444, dgv2682n54, esv2446886, esv2387704, dgv298e199, nsv818921, nsv515833, nsv438218, nsv476769, nsv524627, esv2745990, esv2745991, esv2494968, esv2745992, esv2089729, esv2939, esv2745993, esv2586961, nsv958674, esv997045, esv1020820, nsv471983, nsv52978, nsv973085, nsv748, esv2745994, nsv559205, nsv469458, nsv510299, esv3306300, esv3389043, nsv832445, esv3432454, esv2745995, nsv952197, nsv522671, nsv749, nsv973086, nsv513363, esv1479608, nsv750, nsv751, nsv752, nsv753, esv23857, nsv826405, nsv826406, nsv510300, nsv983341, nsv475563, dgv97n21, nsv515887	chr13:49837894, chr13:48900512, chr12:112104733, chr3:101311470

esv26589, nsv973132, nsv913, nsv975535, nsv517200, nsv952498, nsv560490, esv2746501, nsv52669, esv1005186, esv1123331, nsv52581, nsv7239, esv3413472, nsv513373, esv2660091, dgv22n68, nsv560491, nsv455732, nsv560492, nsv914, nsv560493, nsv560494, nsv476743, esv2646329, esv2761773, esv28503, esv996114, nsv826532, esv1057986, esv3338866, esv2746502, esv2746503, esv2746504, nsv477316, nsv826533, esv2667356, nsv826534, dgv44e19, esv2746505, esv2417445, esv3412579, esv2677110, esv2659767, esv2746506, esv987770, esv1517115, esv1328844, dgv927e59, nsv952499, esv2746507, esv3409514, esv1489716, esv2746509, esv2422416, nsv560495, esv24540, nsv976655, esv2656660, esv3385376, esv2759918, nsv442292, esv35148, nsv524660, dgv2926n54, nsv455733, nsv832534, nsv519344, esv2619454, esv1436194, esv3332718, esv2746510, esv2746511, esv2274657, esv2746512, esv993337, nsv480736, nsv522321, nsv915, nsv560499, nsv509484, esv2746513, esv2746514, dgv2927n54, esv29401,	chr12:123178353, chr20:32831280, chr12:76451743, chrUn_gl000212:127273, chr2:91934335, chr1:227812214, chr12:7333743

nsv455914, nsv561965, nsv821672, dgv3204n54, dgv3207n54, nsv561979, dgv3211n54, nsv562067, esv21584, esv2661680, dgv3213n54, dgv3214n54, dgv3215n54, dgv3216n54, nsv832635, nsv507707, esv3431288, nsv9082, esv24073, dgv3217n54, nsv562086, nsv562088, nsv524318, nsv826682, nsv983600, esv2672796, esv2761796, nsv512324, esv995936, esv7260, esv4726, esv2747544, esv3248385, esv2665820, nsv826683, esv2747545, nsv519044, nsv1072, nsv832637, esv2747546, esv1222385, esv2747547, esv2747548, esv2663265, esv2747549, esv2751147, esv3374750, esv3309795, dgv227n27, dgv3218n54, esv2747550, esv7546, nsv562091, nsv455918, nsv471140, esv3429513, esv2759942, esv2667656, esv2674703, nsv442315, esv2760321, nsv525504, esv2449958, esv3304456, esv3426759, nsv826684, esv3302842, esv34387, nsv473299, esv2677039, nsv832638, nsv521036, nsv9083, nsv976129, nsv455920, nsv562092, nsv826685, nsv951893, esv2662843, esv3329730, esv3303716, esv3309567, esv3439869, nsv562093, nsv455921, nsv562094,	chr3:12198608, chr6:48116220, chrX:139114811, chr1:113992563, chr15:57538771, chr1:81892451, chr12:52105859, chr10:44284310, chr1:54440282, chr4:83049988

nsv562927, nsv456091, nsv562950,
nsv957481, esv3447529, nsv523519,
esv3402039, nsv477538, nsv521902,
esv3377620, esv8421, esv3411800, nsv1145,
dgv1101e59, nsv456092, nsv562951,
esv2637065, esv3306019, esv3308223,
esv3358385, esv1164001, nsv527215,
esv34003, esv2663767, esv2747873,
dgv237e201, nsv957479, nsv951910,
nsv510934, esv3351822, nsv528508,
nsv519296, esv2761814, nsv832696,
esv2747875, nsv510360, nsv821675,
esv2668346, dgv345n67, esv996074,
nsv826757, nsv562952, nsv832697,
nsv478372, nsv510622, dgv346n67,
dgv347n67, nsv826766, nsv456093,
nsv562953, nsv562954, nsv1146, esv2672999,
esv24692, esv2747876, nsv509512,
esv995962, nsv513395, esv3363961,
nsv832698, esv22911, nsv474342, nsv473298,
esv2747877, nsv977272, nsv526668,
esv3308710, esv3364634, esv2671626,
esv2668656, esv2634464, esv2747878,
nsv7247, esv2490241, esv2747879,
nsv523329, nsv518301, esv2669958

nsv833752, nsv519802, esv1464086, esv2673946, nsv953975, esv2718178, esv2718179, nsv819522, nsv819321, esv3388355, nsv960784, nsv509721, esv3416229, esv991647, nsv960785, esv2763196, nsv513515, esv2464355, nsv478794, nsv819872, nsv960786, nsv960787, nsv525966, esv2718180, esv2665155, esv27676, dgv6267n54, nsv960788, nsv522659, esv22040, nsv518441, nsv960789, esv275168, esv33306, nsv960790, nsv578618, nsv458356, esv2718181, esv2147526, esv2718182, esv2718183, nsv954679, esv1213055, nsv960791, nsv578619, esv2718185, esv2664517, nsv482214, esv2718186, esv2718187, esv2660126, nsv482215, nsv2417, dgv623e199, esv2718188, esv23756, dgv6268n54, dgv6269n54, esv2662395, esv3096012, nsv507887, esv2718189, nsv474554, nsv963004, nsv978771, esv3415797, nsv963005, esv2718190, esv2668294, nsv819366, nsv828437, nsv828438, esv2718191, nsv978772, nsv524284, nsv961177, nsv833753, nsv138142, nsv961178, esv2665806, esv1002859, nsv963006, esv2662307,	chr16:51679669, chr7:87150717, chr1:241082928, chr6:5609121, chr5:79654559, chr9:4944336, chr11:74065472, chr15:66108252, chrX:100142287, chr2:41383691, chr8:142469116, chr18:29991839, chr8:83203525, chr3:39376428, chr2:201927752, chr4:74803992, chr8:82719947, chr13:21522091, chr12:9179790, chr15:67920129, chr7:84612812, chr19:42333659, chr17:2210055, chrX:118354685, chrX:73692514, chr1:191114829, chr4:78908686, chr9:91025121, chr2:207283697, chr1:179425027, chr3:42930797, chr1:202880875, chr13:53216567, chr2:63978489, chr5:153716696, chr15:99824500, chr9:128357914, chr2:195052067, chr10:30806212, chr15:35692832, chr3:80265394, chr1:55002442, chr1:58513524, chr4:24773099, chr2:175174421, chr18:70285442, chr11:110658437, chr6:32292957, chr1:116399252, chr12:93277488, chr2:33861509, chrX:100110016, chr1:80916503, chr5:135764581, chr6:27491225, chr6:166748054, chr4:73672957, chr11:5592229, chr3:147883871, chr19:11960603, chr19:12000032, chr19:12028900, chr19:11927167, chr19:12001437, chr19:12066459, chr19:12034965, chr19:11927200, chr19:11961051, chr19:11953540, chr19:11997678, chr19:11982695, chr20:30757558, chr12:99188231, chr14:67227225, chr18:49138262, chr19:12536369, chr19:12496799

Genes

Microarray Nomenclature

C16orf70, B3GNT9, TRADD, FBXL8, HSF4, NOL3, KIAA0895L, EXOC3L1, E2F4, ELMO3, MIR328, LRRC29, TMEM208, FHOD1, SLC9A5, PLEKHG4, KCTD19, LRRC36, TPPP3, ZDHHC1, HSD11B2, ATP6V0D1, AGRP, LOC100505942, FAM65A, CTCF, CARMIL2, ACD, PARD6A, ENKD1, C16orf86, GFOD2, RANBP10, TSNAXIP1, CENPT, THAP11, NUTF2, EDC4, NRN1L, PSKH1, CTRL, PSMB10, LCAT, SLC12A4, DPEP3, DPEP2, LOC100131303, DDX28, DUS2, NFATC3, ESRP2, MIR6773, PLA2G15, SLC7A6, SLC7A6OS, PRMT7, SMPD3, ZFP90, CDH3, CDH1, MIR7641-2, TANGO6, HAS3, CHTF8, UTP4, SNTB2, VPS4A, PDF, COG8, NIP7, TMED6, TERF2, CYB5B, MIR1538, NFAT5, NQO1, NOB1, WWP2, MIR140, CLEC18A, PDXDC2P, MIR1972-2, MIR1972-1, PDPR, LOC400541, CLEC18C, LOC105371328, SMG1P7, EXOSC6, AARS, DDX19B, LOC100506083, DDX19A, ST3GAL2, FUK, COG4, SF3B3, SNORD111B, SNORD111, IL34	arr[hg19] 16q22.1(67,174,211-70,676,534) hmz

CLUHP3, ZNF720, ZNF267, LOC102723753, HERC2P4, TP53TG3D, LOC390705, TP53TG3, TP53TG3B, TP53TG3C, SLC6A10P, ENPP7P13, LINC00273, UBE2MP1, LINC01566, FRG2DP, TP53TG3HP, FLJ26245	arr[hg19] 16p11.2p11.1(31,630,502-35,220,544) hmz

Genes	Microarray Nomenclature
SPATA21, NECAP2, CROCCP3, MIR3675, NBPF1, CROCCP2, MST1P2, ESPNP, MST1L, CROCC, MFAP2, ATP13A2, SDHB, PADI2, PADI1, PADI3, PADI4, PADI6, RCC2, ARHGEF10L, ACTL8	arr[hg19] 1p36.13(16,736,737-18,176,173) hmz

PPP2R3A, MSL2, PCCB, STAG1, SLC35G2, NCK1, IL20RB	arr[hg19] 3q22.2q22.3(135,285,824-136,690,391) hmz

XXYLT1, XXYLT1-AS2, ACAP2, MIR5692C1, PPP1R2, APOD, SDHAP2, MIR570, MUC20, MUC4, TNK2, SDHAP1	arr[hg19] 3q29(194,650,488-195,753,451) hmz

arr[hg19] 4p15.1(32,417,743-34,539,188) hmz

MOB1B, DCK, SLC4A4, GC, NPFFR2	arr[hg19] 4q13.3(71,709,992-72,972,453) hmz

KIF2A, DIMT1, IPO11, LRRC70, IPO11-LRRC70	arr[hg19] 5q12.1(61,362,329-62,376,568) hmz

NBPF22P, COX7C, MIR3607	arr[hg19] 5q14.3(84,996,731-86,195,913) hmz

SGCD, PPP1R2P3, TIMD4, HAVCR1, HAVCR2, MED7, FAM71B, ITK, CYFIP2, FNDC9, NIPAL4, ADAM19, SOX30, C5orf52, THG1L, LSM11, CLINT1	arr[hg19] 5q33.3(156,038,647-157,296,182) hmz

GTF2IRD1, GTF2I, NCF1, GTF2IRD2, STAG3L2, PMS2P5, GATSL1, WBSCR16, GTF2IRD2B, NCF1C, GTF2IP1, LOC100093631, GATSL2, SPDYE8P, PMS2L2, STAG3L1, TRIM73, TRIM74, NSUN5P1, POM121C, SPDYE5, PMS2P3, HIP1	arr[hg19] 7q11.23(73,843,868-75,167,160) hmz

IMMP2L, LRRN3, DOCK4	arr[hg19] 7q31.1(110,303,526-111,466,461) hmz

MIR1208 arr[hg19] 8q24.21(129,124,948-130,225,683) hmz

SLC35D2, ZNF367, HABP4, CDC14B, AAED1, LOC441455, ZNF510, ZNF782, LOC100132781, LOC441454, NUTM2G, HIATL2, CTSL2, LOC340508, LOC100499484, LOC100499484-C9ORF174, CCDC180, LOC286359	arr[hg19] 9q22.32q22.33(99,066,432-100,163,998) hmz

ENG, AK1, MIR4672, ST6GALNAC6, ST6GALNAC4, PIP5KL1, DPM2, FAM102A, NAIF1, SLC25A25, LOC100289019, PTGES2, PTGES2-AS1, LCN2, C9orf16, CIZ1, DNM1, MIR199B, MIR3154, GOLGA2, SWI5, TRUB2, COQ4, SLC27A4, URM1, MIR219-2, MIR2964A, CERCAM, ODF2, GLE1, SPTAN1, WDR34, SET, PKN3, ZDHHC12, LOC100506100, ZER1, TBC1D13, ENDOG, C9orf114, CCBL1, LRRC8A, PHYHD1	arr[hg19] 9q34.11(130,607,711-131,690,509) hmz

ZNF488, RBP3, GDF2, GDF10, PTPN20A, PTPN20B, FRMPD2P1, BMS1P1, BMS1P5, GLUD1P7, FAM25B, FAM25C, FAM25G, LOC399753, FRMPD2, MAPK8, ARHGAP22	arr[hg19] 10q11.22(48,365,294-49,699,016) hmz

PLEKHA1, MIR3941, ARMS2, HTRA1, DMBT1, C10orf120, FLJ46361, CUZD1, FAM24B-CUZD1, FAM24B, LOC399815, FAM24A, C10orf88, PSTK, IKZF5, ACADSB, HMX3, HMX2, BUB3	arr[hg19] 10q26.13(124,165,614-125,182,983) hmz

ANO3	arr[hg19] 11p14.3p14.2(25,377,880-26,543,422) hmz

SRGAP1, C12orf66, C12orf56, XPOT, TBK1, RASSF3, MIR548Z, MIR548C, GNS, TBC1D30, FLJ41278, WIF1, LEMD3, MSRB3	arr[hg19] 12q14.2q14.3(64,434,226-66,149,363) hmz

HCAR3, HCAR1, DENR, CCDC62, HIP1R, VPS37B, ABCB9, OGFOD2, ARL6IP4, PITPNM2, MIR4304, LOC100507091, MPHOSPH9, C12orf65, CDK2AP1, SBNO1, SETD8, RILPL2, SNRNP35, RILPL1, MIR3908, TMED2, DDX55, EIF2B1, GTF2H3, TCTN2, ATP6V0A2, DNAH10, CCDC92, ZNF664, ZNF664-FAM101A	arr[hg19] 12q24.31(123,201,038-124,499,542) hmz

arr[hg19] 13q21.31q21.32(65,352,957-66,563,970) hmz

UBAC2-AS1, UBAC2, MIR548AN, GPR18, GPR183, FKSG29, MIR623, TM9SF2, CLYBL, MIR4306, ZIC5, ZIC2, PCCA	arr[hg19] 13q32.3(99,774,561-101,101,463) hmz

RGL3, CCDC151, PRKCSH, ELAVL3, ZNF653, ECSIT, CNN1, ELOF1, ACP5, ZNF627, ZNF833P, ZNF823, ZNF441, ZNF491, ZNF440, ZNF439, ZNF69, ZNF700, ZNF763, ZNF433, ZNF878, ZNF844, ZNF788, ZNF20, ZNF625-ZNF20, ZNF625, ZNF136, ZNF44, ZNF563, ZNF442, ZNF799	arr[hg19] 19p13.2(11,509,008-12,539,633) hmz
